# Mechanical experimentation of the gastrointestinal tract: a systematic review

**DOI:** 10.1007/s10237-023-01773-8

**Published:** 2023-11-08

**Authors:** Ciara Durcan, Mokarram Hossain, Grégory Chagnon, Djordje Perić, Edouard Girard

**Affiliations:** 1 Zienkiewicz Centre for Modelling, Data and AI, Faculty of Science and Engineering, Swansea University, Swansea, SA1 8EN UK; 2 Université Grenoble Alpes, CNRS, UMR 5525, VetAgro Sup, Grenoble INP, TIMC, 38000 Grenoble, France; 3 Laboratoire d’Anatomie des Alpes Françaises, Université Grenoble Alpes, Grenoble, France

**Keywords:** Biomechanics, Mechanical characterisation, Mechanical properties, Digestive system, Soft tissues, Constitutive modelling, Finite element analysis

## Abstract

The gastrointestinal (GI) organs of the human body are responsible for transporting and extracting nutrients from food and drink, as well as excreting solid waste. Biomechanical experimentation of the GI organs provides insight into the mechanisms involved in their normal physiological functions, as well as understanding of how diseases can cause disruption to these. Additionally, experimental findings form the basis of all finite element (FE) modelling of these organs, which have a wide array of applications within medicine and engineering. This systematic review summarises the experimental studies that are currently in the literature (*n* = 247) and outlines the areas in which experimentation is lacking, highlighting what is still required in order to more fully understand the mechanical behaviour of the GI organs. These include (i) more human data, allowing for more accurate modelling for applications within medicine, (ii) an increase in time-dependent studies, and (iii) more sophisticated in vivo testing methods which allow for both the layer- and direction-dependent characterisation of the GI organs. The findings of this review can also be used to identify experimental data for the readers’ own constitutive or FE modelling as the experimental studies have been grouped in terms of organ (oesophagus, stomach, small intestine, large intestine or rectum), test condition (ex vivo or in vivo), number of directions studied (isotropic or anisotropic), species family (human, porcine, feline etc.), tissue condition (intact wall or layer-dependent) and the type of test performed (biaxial tension, inflation–extension, distension (pressure-diameter), etc.). Furthermore, the studies that investigated the time-dependent (viscoelastic) behaviour of the tissues have been presented.

## Introduction

**Fig. 1:**
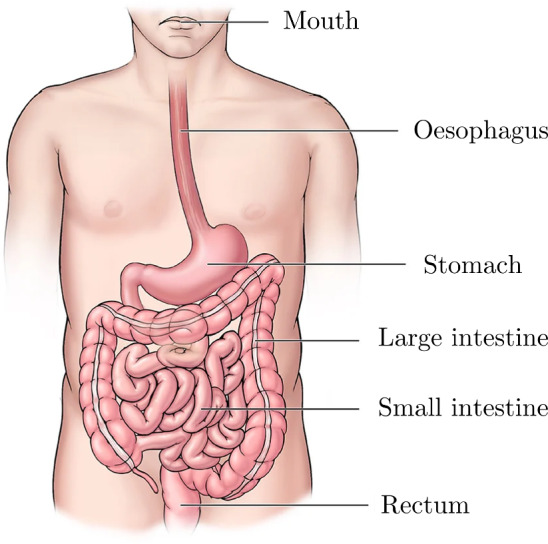
The various organs of the gastrointestinal tract situated in the human body. Figure adapted from Cleveland Clinic (2023)

The gastrointestinal (GI) tract is a muscular tube that extends from the mouth all the way to the anus (Ogobuiro et al. 2021), as can be seen in Fig. 1. The tube is hollow and allows for the passage of food and drink through the body with the aim of extracting its nutrients and expelling the waste products. The oesophagus, the first organ of the GI tract, is responsible for moving the food from the mouth to the stomach. The stomach is responsible for temporarily storing the food, breaking it down both mechanically and chemically and passing it onto the small intestine. The small intestine is the site where 90% of the absorption of nutrients from the food takes place, after which the remaining material is passed onto the large intestine. The large intestine absorbs water and electrolytes from the remaining material. The rectum then stores the solid waste product before expelling it through the anus (Ogobuiro et al. 2021). Each tissue has a slightly different microstructural composition, evolved for the specific function of each organ; for example, villi in the small intestine greatly increase its internal surface area for increased efficiency of nutrient absorption and digestive secretion (Helander and Fändriks 2014). However, all the GI organs have an innermost mucosal layer, an adjacent submucosal layer, then a muscular layer, named the muscularis propria, and, finally, an outermost adventitial (for the oesophagus) or serosal (for the stomach, small intestine, large intestine and rectum) layer. The mucosal layer also contains a thin, muscular layer called the muscularis mucosae (Wanamaker and Grimm 2004). Most collagen and elastin of the GI organs are situated in the mucosal, submucosal and outer layers (Van de Graaff 1986; Baidoo et al. 2022; Durcan et al. 2022a). For a more comprehensive outline of the anatomy of the GI organs, readers are referred to Van de Graaff (1986). Due to the alignment of the fibres in the GI tissues (collagen, elastin and muscle), it can normally be seen that their behaviour is anisotropic (Siri et al. 2020; Durcan et al. 2022a), i.e. they present different stress–strain relations depending on the direction in which the tissue is loaded.

Mechanics are innate to the GI tract’s function. The transportation of food and drink through the tract is brought about by peristalsis: a mechanical process, which propels the ingested material, named fluid bolus when in the oesophagus and chyme when in the other GI organs, through sequential contractions of the muscular wall (Van de Graaff 1986). Peristalsis is also responsible for churning in the stomach, which is a form of physical digestion where the food is mechanically broken down rather than chemically such as with enzymes or stomach acid. This mechanical behaviour of the GI wall is brought about through a combination of passive distensions and active contractions, and the interaction of these with the bolus/chyme (Gregersen and Kassab 1996). The properties of the wall during the passive distensions (such as elasticity, plasticity, and viscosity) provide the stiffness (degree of force exerted by a material when it is loaded) needed along with the active force of the muscle fibres (contractility) to move the hydrodynamic bolus/chyme during peristalsis. Such passive and active properties are organ-specific, depending on their function. For example, the passive material properties of the rectal wall must possess a certain compliance (opposite of stiffness) to be able to accommodate the changing amount of faecal waste product that is temporarily stored there, while the oesophagus requires a different level of compliance to be able to adjust to various bolus sizes that enter it while not being too great as to hinder its primary goal of transporting the bolus to the stomach. However, diseases can affect the passive and active behaviour of the GI tract, disrupting the role of each organ and leading to complications within a patient’s digestive system. For instance, type-2 diabetes has been found to significantly increase the circumferential stiffness of the oesophageal wall in rats (Zhao et al. 2007).

**Fig. 2:**
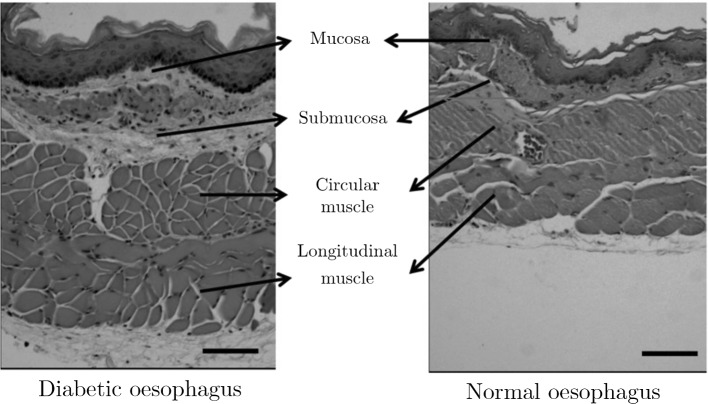
Haematoxylin and eosin (H&E) histological staining of the oesophagus of diabetic Wistar rats (realised through the Goto-Kakizaki (GK) rat model for type-2 diabetes (Goto and Kakizaki 1981)) compared to non-diabetic (normal) Wistar rats, showing the difference between muscle layer thicknesses. The thickness of the longitudinal and circular muscle layers were significantly greater in the diabetic rats compared to the normal rats (*p* < 0.01). Figure has been modified from the review by Zhao and Gregersen (2016) and was originally from a study by Zhao et al. (2007)

From the histological images in Fig. 2, one can see that the onset of diabetes in this animal model has greatly influenced the thickness of the muscularis propria layer, and, as reported by Zhao et al. (2007), has significantly increased the amount of collagen in the mucosa-submucosa layer. These changes in morphology and fraction of microstructural components may allude to the origin of mechanical disorders of the GI tract commonly found in diabetic patients (Horowitz and Samsom 2004); due to the disease, the tissue wall is remodelled and the careful balance of forces that exist in the GI tract between the bolus and the passive/active properties of the wall, that keep the digestive system of so many humans running smoothly, has been disrupted (Frøkjær et al. 2007). Similar biomechanical changes caused by type-1 and type-2 diabetes have been found for other organs of the GI tract including the stomach (Liao et al. 2006), small intestine (Jørgensen et al. 2001; Zhao et al. 2003a) and large intestine (Zhao et al. 2009). Experimentation allows for the investigation into the origin of these disruptions to the GI tract’s mechanical function, providing the information needed to devise creative ways to treat them. As is known within the scientific method, controls, or study of the healthy tissue’s properties, are required to understand the normal function of the GI tissues, thus allowing the effects of the diseases, and potential ways to remedy them, to be properly established.

Another, potentially more advanced, way that allows for the investigation into the mechanisms of how a healthy GI tract functions, and the effect of the changes that occur under pathophysiological conditions, is the use of *in silico* (computational) models. The three types of computational models typically used in the field of GI biomechanics are finite element (FE) analysis (Liao et al. 2006; Panda and Buist 2019), computational fluid dynamics (CFD) (Ferrua and Singh 2010; Palmada et al. 2023) and fluid–structure interactions (FSI) (Toniolo et al. 2023; Zhang et al. 2016). Finite element models provide a numerical approximation of how the tissue or organ behaves mechanically, i.e. structurally, with consideration of its unique geometry and boundary conditions; CFD models allow for the predication of fluid flow through the digestive tract; and FSI provide a means to investigate the interplay between the fluid within and the tissue/organ material structure of the GI tract. Each of the methods has the ability to deliver understanding of the organs’ fluid or structural relations not always possible through experimentation alone (Toniolo et al. 2022), and the structural properties will be focused on in this review. For instance, using a two-layered FE model, Yang et al. (2007a) established why, in a mechanical context, mucosal folds arise within the oesophagus, presenting what would happen to the active tension required of the muscle layer to maintain normal function if these folds were not present. Physiological processes such as peristalsis (Yang et al. 2007b) and the mechanical breakdown of food in the stomach (Skamniotis et al. 2020) can be studied using FE or FSI models to provide insight into which circumstances (e.g., certain wall thickness, amount of collagen, etc.) lead to in-optimal function (Panda and Buist 2019). In addition, structural computational models can be used to establish how the organ responds when medical devices are introduced, either to assess the mechanical effects of traditional devices such as endoscopes (Lin et al. 2020), or to aid with the design of novel medical devices such as stents (Peirlinck et al. 2018; Shanahan et al. 2017), capsule endoscopes (Kim et al. 2007; Gao et al. 2010), capsule biopsy devices (Ye et al. 2019) and surgical staples (Nováček et al. 2012; Guo et al. 2021). Used in this way, models can help save time, biological test specimens and other resources needed during the design process. Further to this, FE models can be used to investigate the effects of surgical interventions, such as bariatric surgery (e.g. reduction in the size of the stomach through a partial gastrectomy) used in the treatment of patients with obesity, on the biomechanics of the GI organs (Toniolo et al. 2022), with one aim being to have patient-specific pre- and post-operative computational models of the organ prior to the procedure to provide a means to assess the best surgical intervention and predict potential post-procedural complications. Moreover, surgical simulations are a growing technology which can utilise FE models to provide haptic force feedback information to a surgeon (Chakravarthy et al. 2014), allowing them to practise and hone their skills before conducting surgery on a patient (Badash et al. 2016). In essence, computational models allow us to predict and numerically assess the complex mechanical behaviour of the GI organs under a wide variety of conditions and thus have valuable applications throughout engineering and medicine.

The equations underpinning the type of FE models mentioned above, as well as the structure portion of FSI models, are conservation and constitutive laws, which describe the mechanical behaviour of the tissue according to Newton’s principles and the individual composition of the material, respectively (Patel et al. 2022). Constitutive laws, originating in this case from the domain of continuum solid mechanics, provide a mathematical representation of the tissue’s behaviour and are based on the well-informed theory that each component (constituent) of the material contributes to its overall behaviour, and thus, its material response can be modelled through a summation of the behaviour of each part. This type of modelling, specifically microstructural based constitutive models, allows for the investigation of the effect of different constituents on the material behaviour of the tissue (Holzapfel and Ogden 2020). Due to the different types of fibres in each of the GI organs, and the differing fractions of mechanically-influential fibres such as collagen and elastin, the individual layers tend to present distinct material behaviour, bearing different loads when forces are applied to the whole tissue structure (Dargar et al. 2019). Due to the soft nature of the GI tissues, which allow easily for large deformations of the organ, the stress–strain response is linear at very small strains but quickly becomes nonlinear when deformed further (Egorov et al. 2002; Rosen et al. 2008; Natali et al. 2009; Christensen et al. 2015; Borsdorf et al. 2021). Therefore, nonlinear elastic laws, rather than linear elastic (which are used for traditional engineering materials such as metals and concrete, or for hard tissue like bone), are often used to describe the behaviour of such tissues (and more modern engineering materials such as polymers) (Holzapfel and Ogden 2006; Chagnon et al. 2015). Additionally, the arrangement of the microstructural components of the tissue, such as collagen and elastin fibres, results in the GI organs exhibiting an anisotropic material response. For this reason, anisotropic constitutive models are often employed when representing the behaviour of the GI tissues. Other, more complex behaviour can also be considered in the constitutive model, such as the time-dependent (viscoelastic) and history-dependent (stress-softening) response of the tissue. Constitutive laws can be used to simulate both the passive and active behaviour of the GI tissues. For a comprehensive review on the constitutive laws used to model the GI tract, readers are referred to Patel et al. (2022).

The parameters, i.e. constants, of the constitutive model are specific to the material in question. This, along with the formulation of the constitutive model based on knowledge of the material’s microstructure and the observed experimental behaviour, distinguishes one material from another for, for example, use in multi-material FE simulations. The parameters also allow for a quantitative comparison between different materials, particularly if the same constitutive law is used. To determine these parameters, the model must be compared with experimental data of the tissue (Weizel et al. 2022). Then, the parameters that provide a mathematical simulation closest to that of the experimental data are determined through an optimisation method (Patel et al. 2022). Different types of experiments are required to establish the various aspects of the material’s behaviour, e.g. active or passive, anisotropic, hyperelastic, viscoelastic, stress-softening. Therefore, to be able to determine the effects of disease on the function of the GI organs (experimentally and *in silico*), to model their constitutive behaviour and further understand the contribution of each component, and to be able to model using the FE method the behaviour of the organ as a function of its geometry and boundary conditions, experimental data are required.

This review paper considers this topic, providing a comprehensive, systematic review of the experimental studies currently available in the literature on the biomechanical behaviour of the GI organs. The articles found in the search are presented for each GI organ in terms of their test condition (*ex vivo* or *in vivo*), the origin of tissue tested (human, rodent, porcine, etc.), type of experiment conducted (uniaxial tension, compression, zero-stress state, etc.), and in terms of whether the direction-dependent and layer-dependent behaviour of the organ was studied. Furthermore, the articles investigating the time-dependent behaviour of the GI organs are shown, and those studying the active or diseased state are mentioned. The proportion of experiments conducted on different species for each GI organ are also illustrated, highlighting, in particular, which organs are lacking experimental data on human tissue. Additionally, the most common experimental techniques to characterise the GI organs are outlined, and the prominence within literature of certain experimental practices, such as preconditioning and the use of a physiological saline solution bath, are displayed. This review aims to bring awareness to the experimental data that exists in regard to the mechanical characterisation of the GI organs and highlight what is currently absent as a call for further experimentation in this area. The information presented here can also be used to direct readers to studies in their particular area of interest, for instance, to provide further understanding or experimental data for their own constitutive and FE modelling.

## Review strategy

The systematic search for this review was carried out using the PubMed database. The search was conducted using key terms associated with biomechanical experimentation, such as “biomechanical”, “mechanical”, “properties”, “behaviour”, “response”, “stress”, “strain”, that could be found in the title or abstract of an article in combination with terms for each of the organs studied: oesophagus, stomach, small intestine, large intestine and rectum. The terms used for each organ can be found in Table 1. Even though the rectum is part of the large intestine; it has been treated as a separate organ here due to its unique function in comparison with the remaining large intestine; the rectum is responsible for the storage and excretion of faeces, whereas the other regions of the large intestine absorb water and electrolytes from the consumed material. The results of the search for each organ were then screened according to certain criteria; these included articles published in peer-reviewed journals, i.e. no pre-prints or conference proceedings, that provided novel (original) experimental data on the macrostructural mechanical properties of the organs in question, in particular experimental data that presented/allowed for the establishment of the stress–strain relations of the tissue or provided the pressure–volume relationship of the organ structure. Experimental studies on the sphincters of the GI tract were not included. There was no lower date limit for the articles; however, studies available online after 15 October 2022 were not included. Any articles not retrieved from the search but known by the authors were added to the pool of articles included in this review.

**Table 1:** Search terms specific to each organ of the GI tract, including the Boolean operators used in the systematic search

Organ	Search terms
Oesophagus	Oesophagus OR oesophageal OR esophagus OR esophageal
Stomach	Stomach OR (gastric AND tissue)
Small intestine	Small intestine OR duodenum OR jejunum OR ileum
Large intestine	Large intestine OR colon OR sigmoid OR cecum
Rectum	Rectum OR rectal

## Experimental techniques

A variety of techniques are used to mechanically characterise the GI tissues. The type of test chosen should be in line with the proposed research question, e.g. are physiological or supraphysiological loading conditions more suitable to quantify the material properties of the GI tissues in the setting/application that we are interested in? In this section, we will outline some of the most common experimental techniques used to quantify the biomechanical behaviour of the GI tract.

For the interpretation of data obtained from such experimental techniques, it is commonly assumed that tissues of the GI tract are incompressible. That is to say that during experimental loading, the volume of the tissue does not change (Nolan and McGarry 2016). While this, physically, is not completely true, the high water content of soft tissues means that they often exhibit properties close to incompressibility (Gilchrist et al. 2014); therefore, the assumption is sufficient in producing meaningful results and is valuable in that it provides a simplification that reduces computational cost.

Mechanical experimentation of human or animal soft tissues can be separated into three categories: *in vivo*, *in situ* and *ex vivo*. *In vivo* experimentation is carried out in the natural environment of the organ, while the human/animal is still living. For organs such as the skin, these experiments can be conducted on the surface of the body. However, for the GI organs, as they are inside the body, a device must be inserted into the body to obtain biomechanical measurements. *In situ* tests are those conducted, whilst the tissue is still connected to the body but is not in its completely natural state, such as experiments conducted on an organ accessed via a surgical opening to the chest. *In situ* experiments can be carried out both while the human/animal is alive or post-mortem. *Ex vivo* (sometimes called “*in vitro*”, although “*ex vivo*” is technically more accurate in regard to the macromechanical characterisations of soft tissues) experimentation is when the organ is removed via dissection from its natural environment and, thus, is no longer alive during the mechanical tests. Tissue can be taken from either alive or deceased subjects, however when the tissue is tested, it is always deceased. Firstly, we will describe the *ex vivo* experimental techniques commonly used to characterise the GI tissues, and secondly, we will summarise the *in vivo* techniques. *In situ* tests are the same as those used for either *ex vivo* or *in vivo* experimentation and therefore have not been given their own section.

### Ex vivo

*Ex vivo* experiments are those performed on naturally grown tissues taken outside of their physiological environment, i.e. excised via dissection from alive or deceased subjects. When the experiments are conducted, the tissue is deceased; therefore, measures should be taken to test the tissue as soon as possible to reduce the time-dependent effects of death, such as ischaemia, on the mechanical properties of the tissue (Marie et al. 2019). In addition, measures are also taken within the test set-up to simulate a more physiological environment in terms of moisture, temperature and, sometimes, carbon dioxide and oxygen concentrations (Liu et al. 2017a; Sokolis et al. 2011), reducing these factors as ones that can cause a discrepancy between *in vivo* and *ex vivo* material behaviour (as *in vivo* is often the environment of interest).

#### Uniaxial tension

**Fig. 3:**
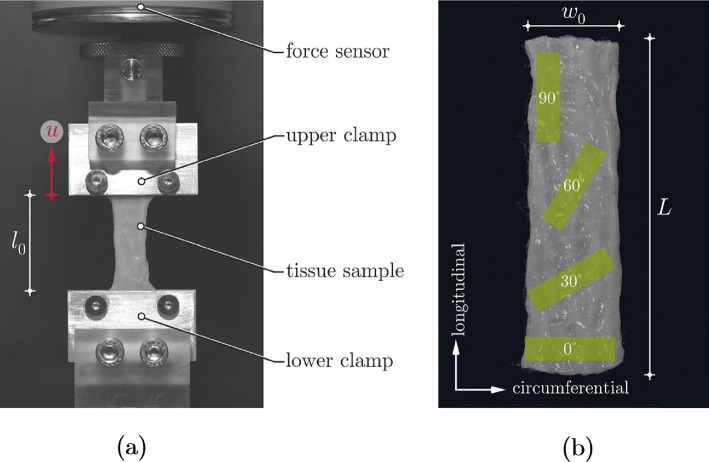
A uniaxial tensile test experimental set-up used to investigate the small intestine of pigs. The bottom clamp (grip) is fixed, while the upper clamp is moved in a displacement-controlled way **a**. Sample preparation of strips of small intestinal tissue for uniaxial tensile testing; to investigate anisotropy (direction-dependent behaviour) of the tissue, specimens can be cut in the longitudinal and circumferential directions, as well as at various angles **b** (Nagaraja et al. 2021)

Uniaxial tensile tests are the most basic tension test in which a specimen of a planar material is loaded along its length, often until failure. For a uniaxial tensile test, the specimen must have a length-to-width ratio of at least 4:1 (ASTM International 2016) (which can be an issue when working with small organs such as the rabbit oesophagus (Jensen et al. 1987)), and the specimens can either be dogbone-shaped (Yang et al. 2006c; Sommer et al. 2013) or rectangular, as seen in Fig. 3. Dogbone samples are more ideal as they encourage rupture to take place in the middle of the specimen rather than at the grip (though this is not guaranteed, and specimens that rupture at the grip should be discarded from analysis); however, it can be difficult to punch consistent dogbone specimens from soft tissues and so in the field of soft tissue biomechanics, it is common to use rectangular-shaped specimens (Egorov et al. 2002; Zhao et al. 2005; Gao et al. 2008; Carniel et al. 2014a; Christensen et al. 2015; Ivakhov et al. 2020).

Uniaxial tensile tests are commonly employed for isotropic materials, such as some metals and polymers (Khan and Liu 2012; Mehnert and Steinmann 2019); however, they can be used to study the anisotropy of a GI tissue by testing strips from the longitudinal (axial) and circumferential directions, and also from various angles in-between these two directions, as seen in Fig. 3b. They cannot, however, be used to determine the radial stress–strain relation of the tissue. Often the grips used to secure the tissue for uniaxial tensile testing have serrated edges or sand paper attached to their inner surfaces to prevent the sample from slipping during testing (Davis et al. 2018; Nagaraja et al. 2021). Sometimes the grips are tightened to a pre-established torque level to find the optimal balance between preventing slippage during testing and not causing the sample to rupture at the grip because they are too tightly secured. Furthermore, tightening the grips to a specific torque provides consistency and reduces the influence of one factor that could affect the repeatability of the results (Davis et al. 2018; Durcan et al. 2022b).

The strain-rate-independent (elastic) behaviour of a tissue can be established under uniaxial tension by loading a sample until failure at a quasi-static strain rate; that is, a strain rate slow enough to allow, theoretically, the viscous relaxation to take place during loading; thus, the material is close to its equilibrium state (material properties once all viscous effects have disappeared). Some experimental studies that perform tests like these precondition their sample first (more on preconditioning in Sect. 4.6.2), removing some of the history- and time-dependent effects that occur during initial loading of a soft biological tissue. Moreover, experiments such as stress-relaxation tests may be carried out to determine the equilibrium stress–strain of the sample (Carniel et al. 2020). Sometimes also called ramp and hold tests, stress-relaxation tests consist of very quickly stretching a sample to a certain strain and holding it there for a considerable amount of time. For soft tissues, it is expected that the stress within the tissue when held will decrease. The length of time that the material is held depends on its relaxation time: for some soft tissues it can take as little as 5 min for the stress to plateau during relaxation (Zhao et al. 2003b; Jia et al. 2015; Carniel et al. 2020), while for some polymers it can take around 30 min (Hossain et al. 2012). When carried out over various stretch levels, the stress after the relaxation period plotted against the strain at which the sample was stretched provides the equilibrium stress–strain relation of the material and, in the context of large strain, can be used to model its hyperelastic behaviour. Creep tests are similar to stress-relaxation tests in that the equilibrium stress–strain relation of the material can be established; however, creep tests are load-controlled rather than strain-controlled. For creep tests, a certain stress is applied to the material and the stress is held at that level while the strain of the sample changes due to viscous effects (Zhao et al. 2003b; Jia et al. 2015). For soft tissues, it can normally be expected that the strain will increase as the sample is held at a certain stress. The maximum deformation (strain) after the creep period can then be plotted against the stress level the sample was held at. Doing this for several stress levels and plotting them on the same graph can provide a picture of the equilibrium stress–strain relationship of the material.

In order to provide a complete picture of the viscoelasticity of a tissue, the time-dependent (viscous) behaviour of the material should be investigated alongside the time-independent properties. The time-dependent behaviour can be studied by conducting uniaxial tensile tests at several different strain rates, including those within and above the quasi-static range and ideally an order of magnitude apart, e.g. 0.1 mm/s, 1.0 mm/s and 10 mm/s (due to the variable nature of soft tissues and thus their mechanical response, an order of magnitude between the strain rates provides a big enough range to be able to experimentally observe the strain rate effects. Tensile tests can also be carried out at dynamic strain rates to establish the behaviour of the tissue under impact. Additionally, cyclic tests can be performed to investigate the differences between the loading and unloading curves. If the sample has been preconditioned, the difference between the loading–unloading curve that remains is thought to be mainly due to the time-dependent relaxation of the specimen. Uniaxial tensile tests are popular in determining the active properties of soft tissues. In this case, the sample is held at zero strain, or other strain levels, and is either activated using a compound, such as potassium chloride, which activates muscle contraction or via electrical stimulation (Jiang et al. 2017; Tomalka et al. 2017). The measured force and change in length of the sample are then used to establish the stress–strain relation under active conditions.

#### Biaxial tension

**Fig. 4:**
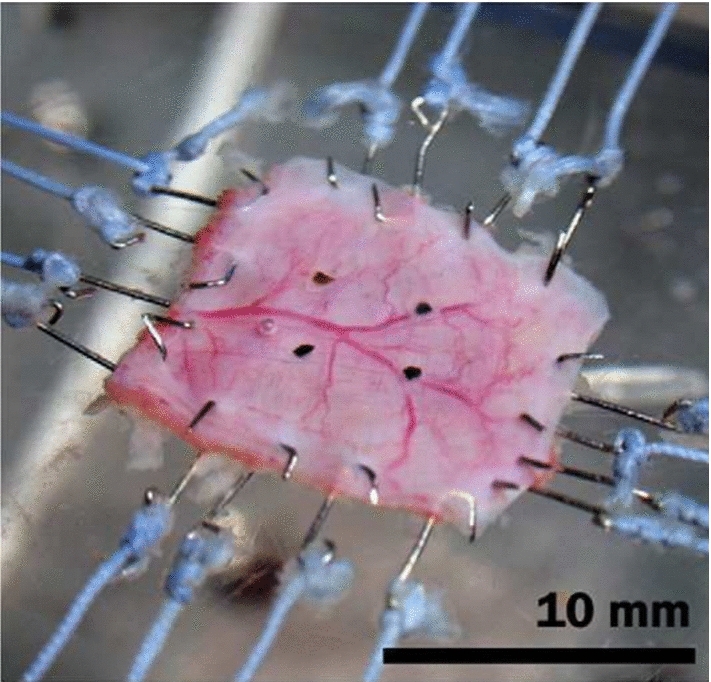
A biaxial tensile test experimental set-up used to investigate the small intestine of pigs. Deformation is applied to a square sample (10 mm × 10 mm) through hooks attached to each side. Four graphite markers were placed on the surface of the sample to optically track its displacement during testing (Bellini et al. 2011)

Biaxial tensile tests are similar to uniaxial tensile tests in that they are performed on planar materials under tension; however, biaxial tests consist of stretching a square sample of a material along two orthogonal directions simultaneously, as seen in Fig. 4; hence, with each individual tissue sample, biaxial tests allow the direction-dependent properties of the tissue to be studied. On this note, biaxial tensile tests are often preferred to uniaxial tensile tests in the domain of hollow soft tissue mechanics as, by stretching the tissue in two directions at the same time rather than testing isolated strips in only one direction, biaxial tension is closer to the *in vivo* loading environment of the organ wall. The stretching in two directions can either be to the same degree, which is called equibiaxial tension, or by different amounts per direction. The choice of this will depend on the application, e.g. during physiological loading conditions, the tissue may undergo differing amounts of stretch in the circumferential and longitudinal directions, thus it may be of value to prescribe different amounts of loading to the circumferential and longitudinal directions to match those typically experienced *in vivo*.

For biaxial tension, the samples must be square, but the size is not critical as long as it is well supported by the testing machine (Bellini et al. 2011; Sommer et al. 2013). This freedom with size can be useful in particular for soft tissue specimens where the number of samples available is either often severely limited, e.g. with human testing, or should be kept to a minimum due to ethical considerations, e.g. with animal testing. The square sample size can be adjusted to allow for as many test samples as possible from a single excised organ. As shown in Fig. 4, the gripping mechanism for biaxial tensile tests is different to that for a uniaxial tension system. Here, several hooks placed equidistantly along each side of the square sample are used to secure and then stretch the specimen. When the specimen is set-up, the time-independent and time-dependent behaviour of the tissue can be studied using similar methods for uniaxial tension, e.g. cyclic testing, varying strain rates, stress-relaxation, etc., as outlined in Sect. 3.1.1.

#### Pure shear

**Fig. 5:**
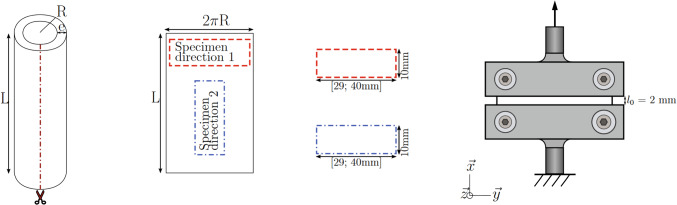
Schematic diagram of planar tension (pure shear) sample preparation and experimental set-up. Figure modified from the work of Masri et al. (2018) on the human male urethra. Although the urethra is not part of the GI tract, it has similar anatomical characteristics and physiological roles as the GI organs in that it is tubular and enacts peristalsis to excrete a waste product (urine). The hashed lines depict a fixed lower grip, while the arrow shows the direction the upper grip moves to apply tension to the sample. Note that Masri et al. (2018) studied the anisotropic properties of the human urethra under planar tension by testing samples in both the longitudinal and circumferential directions

Pure shear tests, sometimes called planar tensile tests, are similar to uniaxial tension tests in that rectangular samples are stretched in only one direction. With pure shear tests, however, the width of the sample is much larger than its length, as can be seen in Fig. 5, for which the length-to-width ratio must be at least 1:2 (Mulvihill and Walsh 2013). This ensures that no significant contraction can take place along the width during loading, making it that the tension in one direction is equal to the orthogonal direction’s compression, producing no rigid body rotation and thus only shear strains within the specimen. Furthermore, pure shear tests are similar to uniaxial tensile tests in that the grips are often serrated or have sand-paper attached to them to reduce slippage of the sample during testing (Davis et al. 2018), and similar tests can be conducted to establish the time-independent and time-dependent behaviour (Sect. 3.1.1).

#### Simple shear

**Fig. 6:**
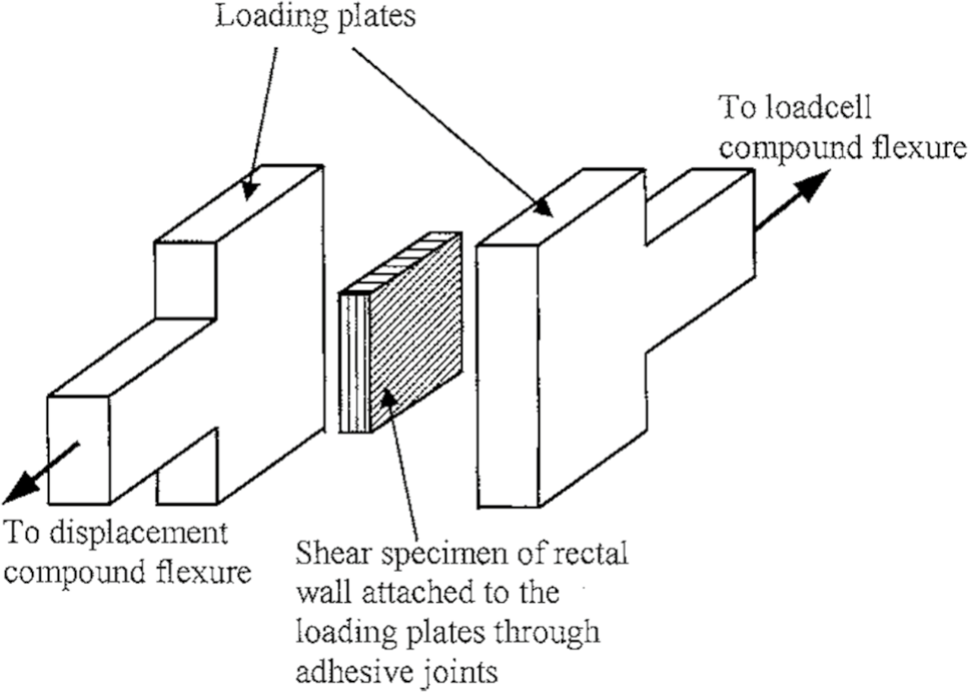
Schematic diagram of a simple shear test being conducted on the rectum from pigs. The arrows indicate the direction the plates move during testing (Qiao et al. 2005)

In the domain of small deformation, simple shear differs from pure shear in that in the simple shear strain state, rotation can occur; this was found to be not fully the case in large deformation; however, as divergence in stress–strain behaviour between the two states can occur at large strain values (Moreira and Nunes 2013). A typical simple shear test set-up is like that which can be seen in Fig. 6, where the sample’s bottom and top surfaces are translated relative to each other. Simple shear tests provide the opportunity to determine the behaviour of the tissue under non-normal forces (those applied parallelly to the tissue surface) as well as the material’s shear modulus, which is useful when considering the types of deformations that exist during normal function of the GI tract (Mishra and Rao 2005).

#### Uniaxial compression

**Fig. 7:**
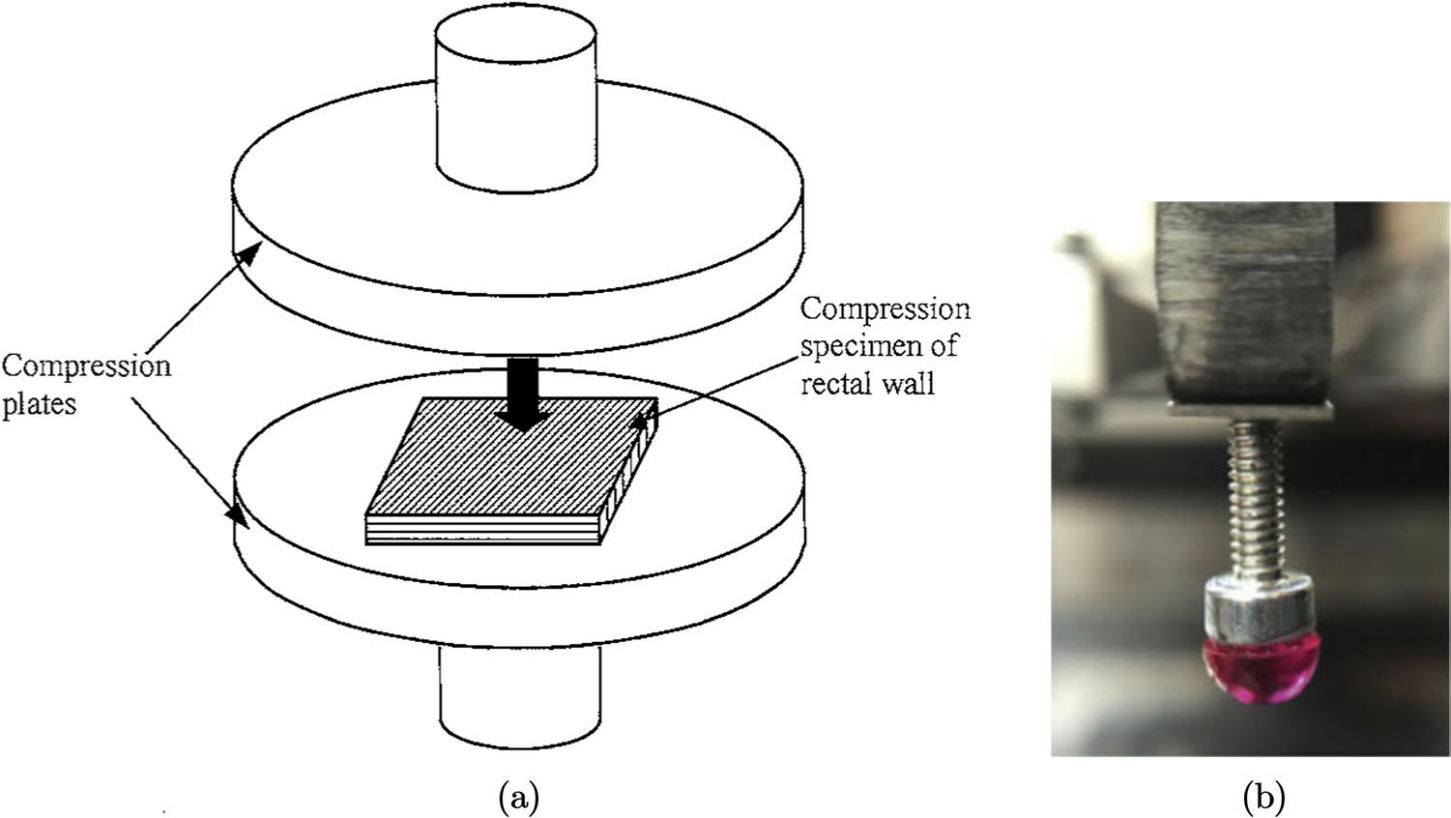
**a** Schematic diagram of a uniaxial compression test being conducted on the rectum from pigs. The arrow indicates the direction the top plate moves during testing (Qiao et al. 2005). **b** Semi-spherical indenter used to investigate the large intestine from rats. The indenter is rigid and has a 3-mm-diameter, which comes into contact with the tissue during testing. **b** modified from Stewart et al. (2016)

Uniaxial compression tests are carried out by pressing a sample of tissue between two plates, as seen in Fig. 7a. These tests involve subjecting a uniform sample, either a square or a short cylinder, to compressive deformation in order to study the behaviour of the tissue and its ability to bear load under compressive strains. The tests used to establish the time-independent and time-dependent behaviour of a soft tissue outlined in Sect. 3.1.1, such as creep, stress-relaxation and cyclic tests, can also be applied to compression tests; however, instead of stretching the material, the applied load will be a compression.

#### Indentation

Similarly to compression tests, indentation tests also prescribe compressive strains to a material; however, the indenter causing the displacement is not a plate covering the entirety of the sample, rather a probe with a compression area that is much smaller than the surface of the sample where the compression is taking place. The shape of the indenter attached the probe can be a more unusual shape compared to the flat plate used for traditional compression testing, for instance a semi-sphere as seen in Fig. 7b, allowing for more nuanced loading regimes (Toniolo et al. 2022). As the only constraints are that the surface where the test takes place is much larger than the size of the indenter and is relatively flat, the tissue specimens for indentation testing can be almost any shape. This is useful for tissues where it can be difficult to cut uniform specimens.

#### Distension

**Fig. 8:**
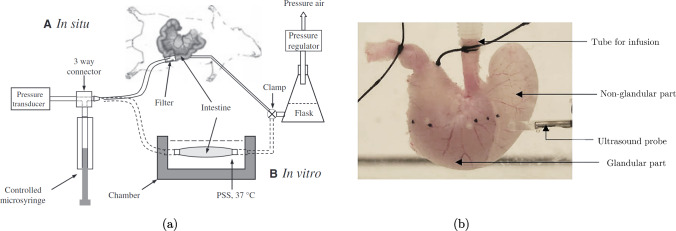
**a** Schematic diagram of both *in vitro*, i.e. *ex vivo*, and *in situ* experimental set-ups for distension testing. Distension and contractility were studied in regard to the small and large intestines of mice (Lu et al. 2012). **b** A distension test experimental set-up used to investigate the stomach of diabetic and non-diabetic rats. A range of luminal pressures were applied to the organ specimen, and the displacements were measured through three-dimensional ultrasound imaging. **b** modified from Liao et al. (2006)

Distension tests, also called inflation tests, for the GI organs, or other hollow organs, involve the stretching of the organ from its inside. A schematic diagram of a distension test conducted both *ex vivo* and *in situ* on the small and large intestines is shown in Fig. 8a, and an example of an experimental set-up for a distension test on the stomach can be seen in Fig. 8b. Note that in Fig. 8a the fluid being injected into one end of the oesophagus flows out the other end of the oesophagus. In this study, the authors recorded the pressure exerted by the fluid on the wall of the organ and measured the intestinal diameter (Lu et al. 2012). Contrarily, the fluid injected into the stomach seen in Fig. 8b is not able to pass out of the other side; for this study, the authors measured the circumferential and longitudinal deformations using three-dimensional ultrasound imaging (Liao et al. 2006). These studies show just two examples of how a distension test can be carried out, in which there are many variations. The essence of the test is the same, however, in that a fluid (liquid or gas) is injected into the hollow organ creating a pressure on the organ wall. The pressure is recorded along with a strain measure (diameter, cross-sectional area (CSA), wall thickness, arc length, three-dimensional imaging) and/or the volume of fluid. Usually *ex vivo* distension tests are performed on passive tissue; however, it is possible to quantify the contractility of the specimen and thus calculate the contribution of the passive and active stress on the organ’s mechanical behaviour.

#### Inflation–extension

**Fig. 9:**
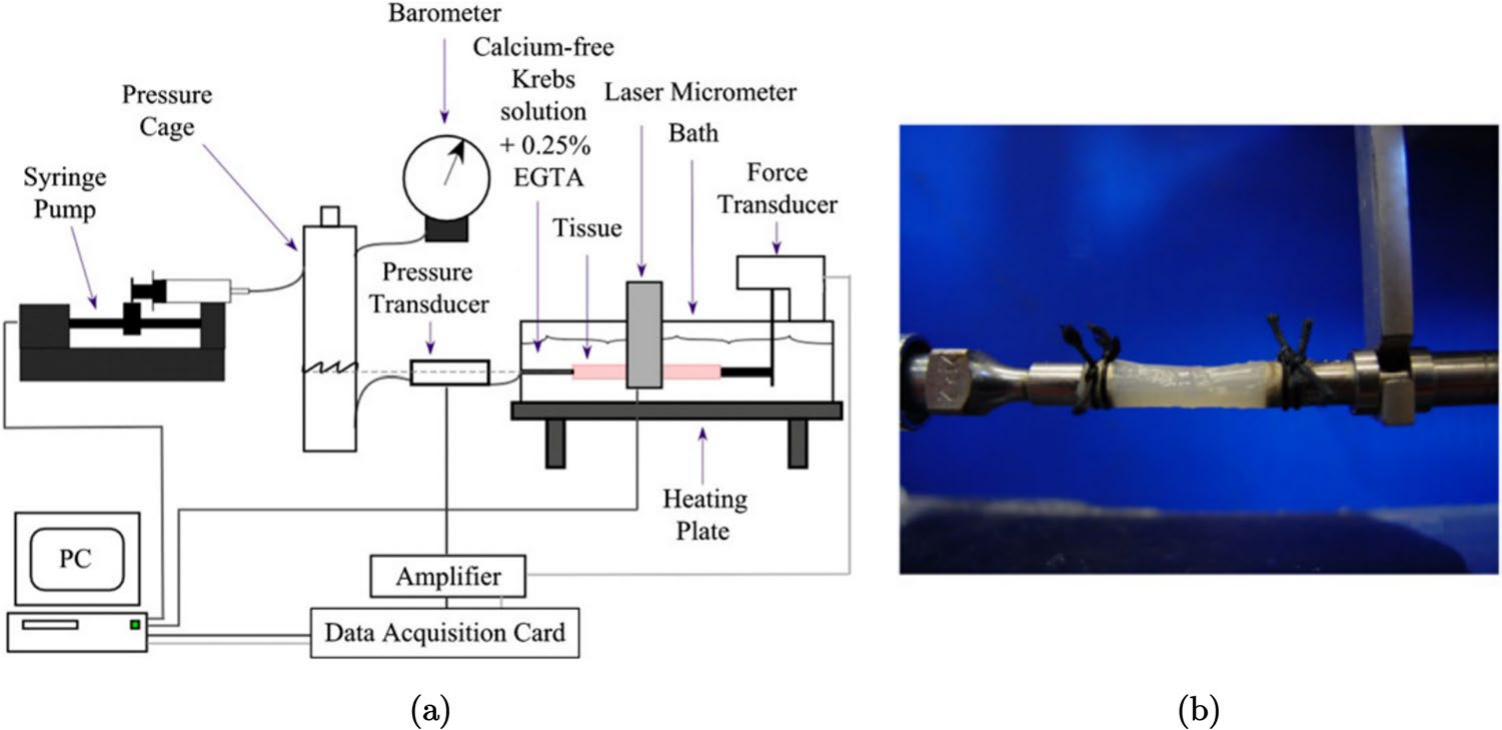
Schematic diagram of an inflation–extension experimental set-up **a** and a close-up of a segment of rodent (Wistar rat) large intestine held in the grips prior to testing **b** (Sokolis et al. 2011)

While distension tests measure the stress–strain relation of an organ in one loading condition, inflation–extension tests measure it in two. Inflation–extension tests, as the name suggests, involve both distension of the tissue in the circumferential direction and stretch in the axial/longitudinal direction, allowing for characterisation of the tissue’s anisotropic properties in a state closer to *in vivo* conditions compared to uniaxial or biaxial tensile testing, i.e. with the organ structure intact. An example of the experimental set-up for an inflation–extension test can be seen in Fig. 9.

#### Zero-stress state

**Fig. 10:**
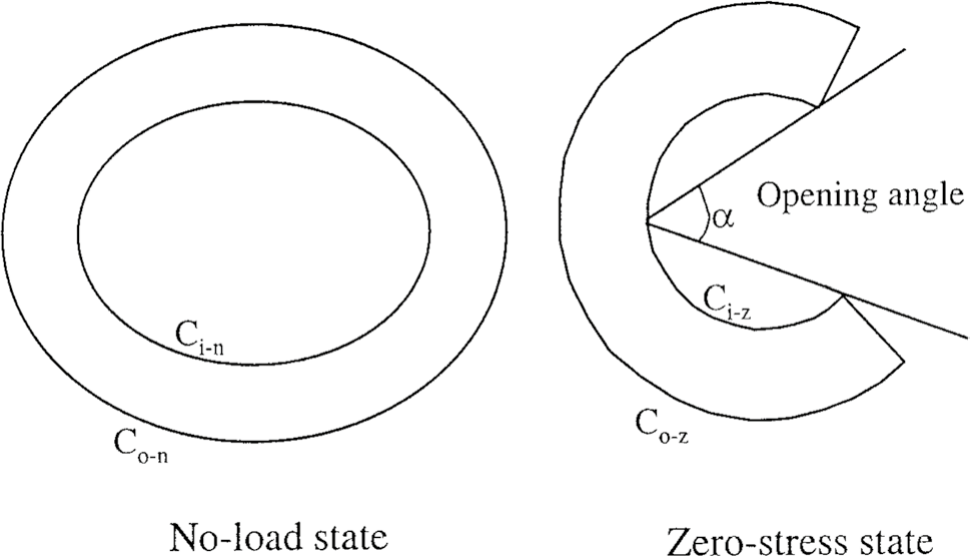
Schematic diagram of how a circumferential ring segment of a residually stressed tubular organ deforms from its no-load state to its zero-stress state, including a schematic definition of the opening angle ($\alpha$) (Gao et al. 2000)

It can be the case that the no-load state of a tissue is different from its zero-stress state. In 1983, both Vaishnav and Vossoughi (1983) and Fung (1984) demonstrated this to be the case with arteries, and since then it has been determined that many other soft biological tissues also possess residual stresses and strains in their no-load configuration, including the GI tract (Gregersen et al. 2000). The purpose of these residual strains has been attributed to providing a more balanced stress distribution within the organ wall (Aggarwal et al. 2016). Figure 10 shows a schematic diagram of how a ring segment of a residually stressed tubular tissue deforms between its no-load state and its zero-stress state; the ring specimen deforms into a sector when, in its no-load state, it is cut radially, producing a parameter by which the degree of residual strains within a tubular tissue can be defined: the opening angle, $\alpha$. The greater the opening angle, the greater the residual strains in the tissue specimen. Therefore, the opening angle can be used to compare the varying degree of residual strains throughout an organ (e.g. along its axial length) or between organs. To determine the residual stresses, however, the residual strains must be quantified. For this, the morphology of the tissue, i.e. the inner and outer circumferences of the different layers within the ring specimens, before and after deforming to the zero-stress state can be used to establish the residual strains present. From here, the residual stresses can be calculated via a constitutive law.

**Fig. 11:**
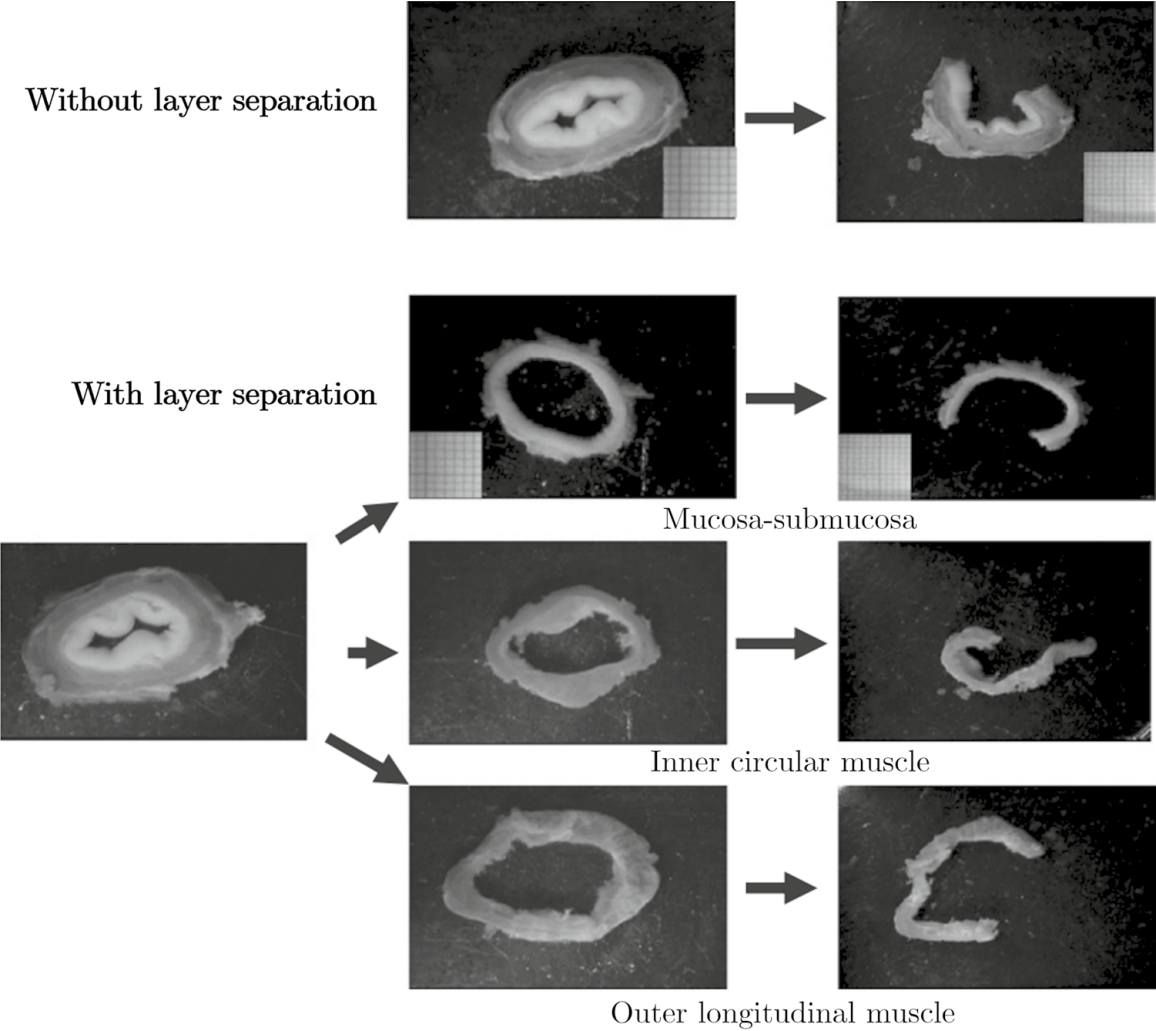
Experimental results showing the no-load and zero-stress state of circumferential ring specimens from the oesophagus of pigs, investigating the residual strains of the intact wall as well as the separated layers (mucosa-submucosa, circular muscle and longitudinal muscle). Figure modified from Zhao et al. (2007)

To determine the circumferential residual strains of a tubular tissue, the usual protocol is that described in Fung and Liu (1989) where ring-like specimens of the tissue, 1–2 mm in length, are cut. The cross-section of these specimens are photographed, as seen in the pictures on the left in and in the centre of Fig. 11, then a radial cut is made to the wall of the ring. Usually this causes the specimens to open into an sector, as seen in Fig. 10 and on the right in Fig. 11. The specimens are given time to equilibrate, allowing any viscous effects to dissipate, and are then photographed again. The difference in lengths of the inner and outer circumferences of the specimens from the closed ring to the open sector are used to calculate the residual strains of the tissue. The closed ring is when the tissue is in the no-load state, i.e. no external loads such as luminal pressures are exerted on the wall, and the open sector is considered the zero-stress state, when all the internal, residual stresses of the material have been released. This method is based on some assumptions such as that the ring specimen is a perfect circle, though in reality this is not often the case. Recently, in 2019 and 2021, respectively, Sigaeva et al. (2019) and Lefloch et al. (2021) developed novel ways of assessing residual strains without this perfect circle assumption to make the measurement of tissue rings more accurate, particularly when the tissue being investigated is diseased (as these specimens are often more irregular compared to healthy tissue). However, within the literature, currently most zero-stress state studies still use the method outlined in Fung and Liu (1989), which is reasonably accurate when the samples keep their mainly circular geometric formation. As can be seen in Fig. 11, this technique can be carried out on intact wall specimens or on ring-like specimens separated into their different composite layers, e.g. the mucosa-submucosa layer and the muscularis propria layer. It is also possible to study how the ring segments open over time, thus including the viscous effects (i.e. time-dependent effects) in the residual stress/strain analysis.

Longitudinal prestretch can be determined by measuring the difference between the length of the tubular tissue *in situ* and comparing this to its length *ex vivo*. In addition, longitudinal strips can be cut free from the wall and allowed to equilibrate. Similarly to the circumferential samples, the deformations of these longitudinal strips can be used to determine the residual strains in the longitudinal direction.

### In vivo

*In vivo* experimentation is that carried out in the organ’s natural environment on a subject which is alive. While *ex vivo* experimentation is often very similar to standard engineering material characterisation tests, *in vivo* tests on the GI organs pose an added complication of needing to measure the deformations of a material, which cannot be seen with the naked eye (Dargar et al. 2016).

#### Distension

Distension tests conducted *in vivo* are similar to those carried out *ex vivo* (Sect. 3.1.7); however, while a balloon is sometimes used when testing *ex vivo*, it is always used *in vivo* in order to keep the fluid contained. Unlike *ex vivo* distension testing, the outer diameter cannot be simply measured using a camera to determine the strain of the sample. Therefore, modalities such as impedance planimetry and ultrasound must be employed to determine the strain of the wall in relation to the pressure exerted by the volume of fluid injected into the organ’s lumen (Drewes et al. 2001; Takeda et al. 2002; Petersen et al. 2003; Takeda et al. 2003).

#### Elastography

Elastography is a technique that can be used to non-destructively determine the mechanical properties of the GI tract *in vivo*, including its layer-dependent properties (Dargar et al. 2019) and thus can be used to quantify a tissue’s material behaviour in its physiological environment (Li and Cao 2017). Furthermore, elastography can be used clinically to identify the health state of soft tissues (Evans et al. 2010; Venkatesh et al. 2013; Kennedy et al. 2017). There are many different types of elastography, and their type depends on how the strains are measured; however, in essence, firstly a stimulus is applied to the tissue, for instance a vibration (Evans et al. 2010) or a compression (Dargar et al. 2019), the deformation is then tracked via an imaging modality such as ultrasound, magnetic resonance or optics, and, finally, the tissue’s mechanical properties are determined computationally through inverse analysis (Li and Cao 2017). For a comprehensive understanding of ultrasound, optical and magnetic resonance elastography, readers are referred to the reviews by Li and Cao (2017), Kennedy et al. (2013) and Low et al. (2016), respectively.

## Review findings

**Fig. 12:**
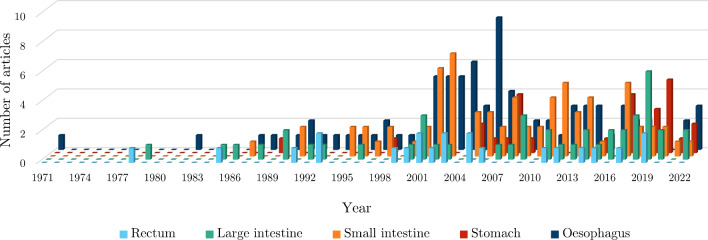
Evolution of the number of articles published per year per GI organ according to this review

**Fig. 13:**
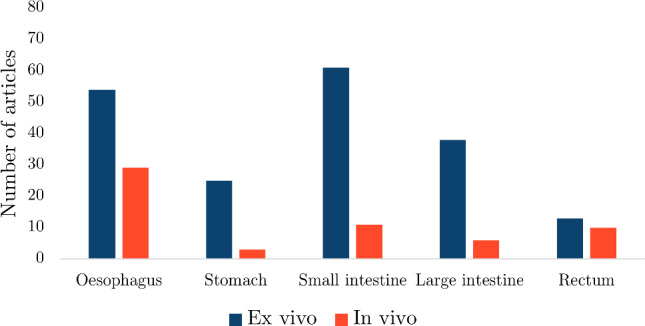
The number of *ex vivo* and *in vivo* studies collected per organ

The number of search results, articles screened from the search and articles added by the authors for each organ can be found in Table 2. Out of all the articles, the proportion of studies collected for the oesophagus was 33%, for the small intestine 29%, for the large intestine 18%, for the stomach 11% and for the rectum 9%. Figure 12 shows the number of publications for each organ as a function of year in which they were published. The results for each organ were organised into whether the experimentation was conducted *ex vivo* or *in vivo*, for which the number of articles for each state can be seen in Fig. 13.

It should be noted that in this review, experiments conducted *in situ* on alive subjects have been considered as *in vivo*, and *in situ* experiments conducted on deceased subjects have been regarded as *ex vivo*. There were so few *in situ* experiments that they did not warrant a results table of their own. This explains how an “indentation test” may be conducted *in vivo* (Table 4); in actuality it was conducted *in situ* while the subject was still alive, i.e. there was still blood flow in the organs.

**Table 2:** The number of search results for each organ, screened articles from the search, articles added by the authors and the total number of articles considered per organ. Altogether, the total number of articles collected was 247

	Oesophagus	Stomach	Small intestine	Large intestine/colon	Rectum
PubMed search results	732	464	556	653	311
Screened articles from search	61	22	61	36	13
Articles added by authors	21	6	11	7	8
Total number of articles	82	28	72	44	21

In some studies, different types of experiments were conducted, either using various techniques, e.g. *ex vivo* inflation–extension and *ex vivo* zero-stress state analysis, and/or different organs, e.g. large intestine and rectum, and/or different species, e.g. pig and human. From this point forward, each test situation (i.e. species, organ and experimental technique) will be treated as separate even if they are presented within the same article, and will, therefore, be referred to as individual “experiments”.

### Oesophagus

The oesophagus had the greatest number of experimental studies out of all the GI organs (Table 2). The experiments conducted on the oesophagus *ex vivo* are summarised in Table 3. Of these studies, several looked into the effects of pathological conditions on the organ’s mechanical properties, including oesophageal varices in rabbits (Jensen et al. 1987; Gregersen et al. 1991), osteogenesis imperfecta in mice (Gregersen et al. 2001), oesophagitis in humans (Vanags et al. 2003), diabetes in rats (Yang et al. 2004; Zeng et al. 2004a, b; Yang et al. 2006; Zhao et al. 2007; Liu et al. 2012; Jiang et al. 2019) and cancer in pigs (Aho et al. 2016a). Zeng et al. (2004b) looked at how diabetes affects the material behaviour of rodent oesophagi over time. As a treatment for diabetic GI disorder, Liu et al. (2012) studied the effect of Tangweian Jianji (a Chinese medicinal compound) on the mechanical properties of the oesophagus in diabetic rats. Others looked at the effects of epidermal growth factor (EGF) to investigate how abnormal growth may affect the function of the oesophagus in rats (Zhao et al. 2003a), while some investigated the effect of ageing on the mechanical properties of the oesophagus in humans (Vanags et al. 2003) and Wistar rats (Gregersen et al. 2004; Zhao and Gregersen 2015).

**Fig. 14:**
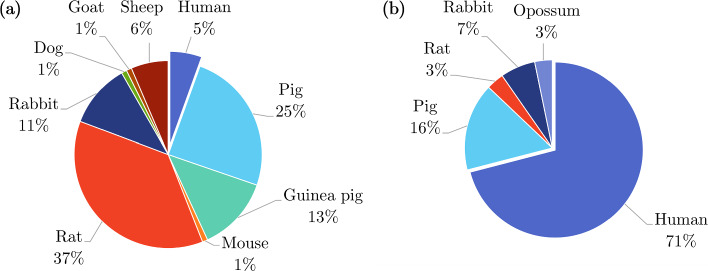
Pie charts indicating the species used in the *ex vivo* experimentation (n=109) **a** and *in vivo* experimentation (n=30) **b** on the oesophagus, highlighting, in particular, the proportion of experiments conducted on human tissue

Most *ex vivo* studies of the oesophagus investigated its passive material properties; however, some studied its active properties: Tøttrup et al. (1990) looked at the active properties of human oesophageal muscle, and Wareham and Whitmore (1982) investigated the active mechanical properties of the muscularis propria of guinea pig oesophagi. As can be seen in Fig. 14a, *ex vivo* experimentation on the oesophagus was conducted using a wide variety of animals. Experiments conducted on oesophagi from rats were the most prevalent, while *ex vivo* experimentation conducted on human tissue accounted for only 5%.

The oesophagus had the most *in vivo* studies of all the organs considered (Fig. 13), a summary of which can be found in Table 4. Several conditions were studied in regard to their effect on the mechanical properties of the oesophagus *in vivo*, including oesophageal varices in rabbits (Gregersen et al. 1991, 1988), nutcracker oesophagus (i.e. abnormal peristalsis) in humans (Mujica et al. 2001), chest pain of oesophageal origin (sometimes referred to as functional chest pain (FCP)) in humans (Rao et al. 2001, 2002; Drewes et al. 2006; Nasr et al. 2010), systemic sclerosis in humans (Villadsen et al. 1997, 2001; Gregersen et al. 2011) and type-1 diabetes in humans (Frøkjær et al. 2007). Gregersen et al. (1992) studied the mechanical changes that occur in the oesophagi of opossums that have been obstructed. Juhl et al. (1994) investigated the effect of damage caused by endoscopic sclerotherapy on the mechanical properties of minipig oesophagi, while Vinter-Jensen et al. (1995) studied the potential viability of EGF as a treatment (therapeutic potential) for this damage, also using oesophagi from minipigs. Drewes et al. (2002, 2003, 2005) conducted several studies on pain perception in relation to distension of the oesophagus in humans. Takeda et al. (2003) studied the active and passive properties of the human oesophagus *in vivo* through the use of a muscle relaxant, atropine. As can be seen in Fig. 14b, the majority of *in vivo* experimentation of the oesophagus was carried out on humans.

**Table 3:** Summary of *ex vivo* studies on the oesophagus

	Species family	Tissue condition	Type of test	References
Isotropic	Human	Intact wall	Uniaxial tension	Egorov et al. (2002)
		Layer-dependent	Uniaxial tension	Tøttrup et al. (1990)
	Porcine	Intact wall	Uniaxial tension	Sanchez-Molina et al. (2014)
			Pure shear	Sanchez-Molina et al. (2014)
			Indentation (dynamic)	Tay et al. (2006)
			Distension (pressure-CSA-wall thickness)	Zhao et al. (2007)
			Inflation–extension	Ren et al. (2021)
			Tribological test	Lin et al. (2017)
			Shear wave vibrometry	Aho et al. (2016a)
			Zero-stress state	Zhao et al. (2007); Ren et al. (2021)
		Layer-dependent	Uniaxial tension	Sanchez-Molina et al. (2014)
			Pure shear	Sanchez-Molina et al. (2014)
			Inflation–extension	Ren et al. (2021)
			Tribological test	Lin et al. (2017)
			Shear wave vibrometry	Aho et al. (2016a)
			Zero-stress state	Yang et al. (2006); Zhao et al. (2007); Ren et al. (2021)
	Ovine	Intact wall	Axial tension of tubular specimens	Saxena et al. (2021)
	Caprine	Layer-dependent	Tension test of ring specimens	Taira et al. (2014)
	Canine	Intact wall	Distension (pressure-diameter)	Badylak et al. (2005)
	Lagomorph	Intact wall	Uniaxial tension	Jensen et al. (1987)
			Axial tension of tubular specimens	Lu and Gregersen (2001)
			Distension (pressure-CSA)	Gregersen et al. (1991); Liao et al. (2006)
			Zero-stress state	Lu and Gregersen (2001); Sokolis (2010)
		Layer-dependent	Zero-stress state	Lu and Gregersen (2001); Stavropoulou et al. (2009); Sokolis (2010)
	Rodent	Intact wall	Distension (pressure-diameter)	Zhao et al. (2003a); Liao et al. (2003); Fan et al. (2004); Zhao et al. (2007); Liao et al. (2009); Jiang et al. (2014, 2019)
			Distension (pressure-CSA)	Assentoft et al. (2000)
			Inflation–extension	Assentoft et al. (2000); Gregersen et al. (2008)
			Axial tension of tubular specimens	Liao et al. (2009)
			Acoustic microsopy	Assentoft et al. (2001)
			Zero-stress state	Gregersen et al. (1999, 2001); Zhao et al. (2003a); Liao et al. (2003); Yang et al. (2004); Gregersen et al. (2004); Zeng et al. (2004a); Yang et al. (2004); Fan et al. (2004, 2005); Yang et al. (2006); Zhao et al. (2007); Gregersen et al. (2008); Liao et al. (2009); Liu et al. (2012); Jiang et al. (2014); Zhao and Gregersen (2015); Jiang et al. (2019)
		Layer-dependent	Uniaxial tension	Wareham and Whitmore (1982)
			Distension (pressure-diameter)	Liao et al. (2003); Fan et al. (2004); Jiang et al. (2017, 2019)
			Acoustic microsopy	Assentoft et al. (2001)
			Zero-stress state	Gregersen et al. (1999); Liao et al. (2003); Fan et al. (2004, 2005); Jiang et al. (2017, 2019)
Anisotropic	Human	Intact wall	Uniaxial tension	Vanags et al. (2003)
			Distension (pressure-diameter-length)	Vanags et al. (2003)
		Layer-dependent	Uniaxial tension	Durcan et al. (2022a, 2022b)
	Porcine	Intact wall	Uniaxial tension	Lin et al. (2019)
			Indentation	Tay et al. (2006)
			Sonometry	Aho et al. (2016b)
		Layer-dependent	Uniaxial tension	Yang et al. (2006a, 2006c, 2006b); Stavropoulou et al. (2012); Lin et al. (2017)
			Inflation–extension	Yang et al. (2006)
			Tribological test	Lin et al. (2019)
			Sonometry	Aho et al. (2016b)
	Ovine	Intact wall	Biaxial tension	Ngwangwa et al. (2022)
			Inflation–extension	Sommer et al. (2013)
			Zero-stress state	Sommer et al. (2013)
		Layer-dependent	Uniaxial tension	Sommer et al. (2013)
			Biaxial tension	Sommer et al. (2013)
			Zero-stress state	Sommer et al. (2013)
	Lagomorph	Intact wall	Inflation–extension	Sokolis (2010, 2013)
		Layer-dependent	Inflation–extension	Stavropoulou et al. (2009)
	Rodent	Intact wall	Torsion	Zeng et al. (2004b)
			Distension (pressure-diameter-length)	Gregersen et al. (2004); Liu et al. (2012); Zhao and Gregersen (2015)
			Distension (pressure-CSA)	Assentoft et al. (2000)
			Inflation–extension	Assentoft et al. (2000)
			Inflation–extension–torsion	Yang et al. (2004); Zeng et al. (2004a); Yang et al. (2004, 2006)
		Layer-dependent	Inflation–extension–torsion	Zeng et al. (2004a); Yang et al. (2006)

**Table 4:** Summary of *in vivo* studies on the oesophagus

	Species family	Tissue characterisation	Type of test	References
Isotropic	Human	Intact wall	Distension (pressure-CSA)	Orvar et al. (1993); Villadsen et al. (1997); Patel and Rao (1998); Mujica et al. (2001); Rao et al. (2001); Villadsen et al. (2001); Takeda et al. (2002); Drewes et al. (2002); Rao et al. (2002); Drewes et al. (2003, 2005, 2006); Nasr et al. (2010); Gregersen et al. (2011); Remes-Troche et al. (2012); Liao et al. (2013)
			Distension (pressure-volume)	Remes-Troche et al. (2012); Mojoli et al. (2016)
			Distension (pressure-CSA-wall thickness)	Takeda et al. (2003)
			Distension (pressure-CSA-volume)	Barlow et al. (2002)
	Porcine	Intact wall	Indentation (dynamic)	Tay et al. (2006)
			Distension (pressure-CSA)	Juhl et al. (1994); Vinter-Jensen et al. (1995); Gregersen et al. (1996)
	Lagomorph	Intact wall	Distension (pressure-CSA)	Gregersen et al. (1988, 1991)
	Marsupial	Intact wall	Distension (pressure-CSA)	Gregersen et al. (1992)
	Rodent	Intact wall	Distension (pressure-diameter)	Goyal et al. (1971)
Anisotropic	Human	Layer-dependent	Distension (pressure-CSA)	Frøkjær et al. (2007)
			Distension (pressure-CSA-volume)	Frøkjaer et al. (2006)
	Porcine	Intact wall	Indentation	Tay et al. (2006)

### Stomach

Only 11% of all the articles collected in this review investigated the mechanical properties of the stomach (Table 2). A summary of the experiments conducted *ex vivo* on the stomach can be found in Table 5. Of these studies, Liao et al. (2006) looked into the effects of disease on the stomach’s material behaviour, in particular the impact of type-2 diabetes on the mechanical properties of stomach tissue from rats. Notably, Carniel et al. (2020) and Toniolo et al. (2022) studied stomach tissue removed from patients (humans) suffering with morbid obesity who had undergone a laparoscopic sleeve gastrectomy, while Marie et al. (2019) investigated how sleeve gastrectomies affect the biomechanical behaviour of the stomach using specimens from pigs for which the surgical procedure had been performed *ex vivo*. In terms of the active behaviour of the stomach, Merlo and Cohen (1989) evaluated the active mechanical properties of its muscle layers with tissue excised from cats, and Tomalka et al. (2017) electrically stimulated the smooth muscle of pig stomachs to assess their behaviour. Furthermore, Klemm et al. (2020) studied both the intact wall of the stomach from pigs (mucosal and muscular layers) and just its muscle layer to determine the contribution of each layer in the tissue’s active behaviour, while Borsdorf et al. (2021) investigated the active response of the combined muscle layer (oblique, longitudinal and circular muscle) and just the circular gastric smooth muscle layer to compare their influence on the mechanical behaviour of the stomach from domestic pigs.

**Fig. 15:**
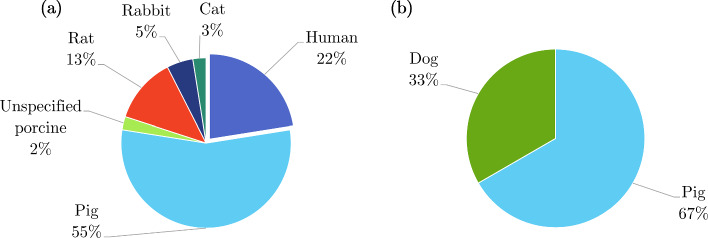
Pie charts indicating the species used in the *ex vivo* experimentation (n=40) **a** and *in vivo* experimentation (n=3) **b** on the stomach, highlighting, in particular, the proportion of experiments conducted on human tissue

*In vivo* experimentation on the stomach was the least common compared to the other GI organs (Fig. 13), for which only the healthy, passive properties were investigated. A summary of the experiments carried out *in vivo* on the stomach can be found in Table 6. Stomach tissue originating from porcine was the overwhelming choice for studying the organ both *in vivo* and *ex vivo*, as can be seen in Fig. 15, with one author stating that this decision originated from “the similarities between the porcine and the human digestive systems” (Salmaso et al. 2020). Only 22% of the *ex vivo* experimentation was performed on human tissue (Fig. 15a), while no human tissue was studied *in vivo* (Fig. 15b).

**Table 5:** Summary of *ex vivo* studies on the stomach

	Species family	Tissue condition	Type of test	References
Isotropic	Human	Intact wall	Simple shear (dynamic)	Saraf et al. (2007)
			Indentation	Lim et al. (2009); Toniolo et al. (2022)
			Confined compression (dynamic)	Saraf et al. (2007)
			Distension (pressure-volume)	Carniel et al. (2020); Toniolo et al. (2022)
	Porcine	Intact wall	Indentation	Rosen et al. (2008)
			Distension (pressure-volume)	Fontanella et al. (2019); Marie et al. (2019); Salmaso et al. (2020)
		Layer-dependent	Uniaxial tension	Tomalka et al. (2017); Ivakhov et al. (2020); Borsdorf et al. (2021)
			Indentation	Kunkel et al. (2008)
			Compressive elastography	Dargar et al. (2016)
			T-peel	Ivakhov et al. (2020)
	Feline	Layer-dependent	Uniaxial tension	Merlo and Cohen (1989)
	Rodent	Intact wall	Distension (pressure-CSA)	Liao et al. (2004)
Anisotropic	Human	Intact wall	Uniaxial tension	Egorov et al. (2002); Carniel et al. (2020); Toniolo et al. (2022)
	Porcine	Intact wall	Uniaxial tension	Gao et al. (2008); Zhao et al. (2008); Jia et al. (2015); Marie et al. (2019); Klemm et al. (2020)
			Uniaxial tension (dynamic)	Julie et al. (2022)
			Biaxial tension	Aydin et al. (2017); Bauer et al. (2020)
		Layer-dependent	Uniaxial tension	Zhao et al. (2008); Jia et al. (2015); Klemm et al. (2020)
			Biaxial tension	Bauer et al. (2020)
			Pure shear	Davis et al. (2018)
	Lagomorph	Intact wall	Uniaxial tension	Zhao et al. (2005)
			Zero-stress state	Zhao et al. (2005)
	Rodent	Intact wall	Uniaxial tension	Zhao et al. (2005)
			Distension (pressure-volume)	Zhao et al. (2005); Liao et al. (2006)
			Zero-stress state	Zhao et al. (2005)

**Table 6:** Summary of *in vivo* studies on the stomach

	Species family	Tissue characterisation	Type of test	References
Isotropic	Porcine	Intact wall	Indentation	Rosen et al. (2008)
	Canine	Intact wall	Distension (pressure-volume)	Shafik (1998)
Anisotropic	Porcine	Layer-dependent	Compressive elastography	Dargar et al. (2019)

### Small intestine

Of all the GI organs, the majority of *ex vivo* experimentation was conducted on the small intestine (Fig. 13). The summary of *ex vivo* experiments on the small intestine can be found in Table 7. Conditions affecting the small intestine were studied, including diabetes in rats (Jørgensen et al. 2001; Zhao et al. 2002, 2003a, b, 2013a, 2017), intestinal oedema in rats (Radhakrishnan et al. 2005, 2006), and Chinese medicines, namely Kaiyu Qingwei Jianji (Sha et al. 2006) and Tangweian Jianji (Liu et al. 2012), were investigated in rats regarding their ability to treat the GI symptoms associated with diabetes. In addition, Zhao et al. (2013b) investigated the active mechanical properties of the small intestine from rats with diabetes and rats with a condition that mimics human irritable bowel syndrome (IBS) (Zhao et al. 2019). The effects of clinical interventions on the mechanical properties of the small intestine were also studied, including irradiation as a treatment for jejunal fibrosis in mice (Peck and Gibbs 1987), chronic coeliac ganglionectomy in rats (Ouyang et al. 1996), small intestinal resection in rats (Dou et al. 2002a) and distraction enterogenesis in pigs (Hosseini and Dunn 2020).

The influence of growth on the mechanical behaviour of the small intestine was evaluated naturally, i.e. during physiological growth, in rats (Lu et al. 2005) and using EGF (Vinter-Jensen et al. 1996; Zhao et al. 2002; Liao et al. 2003; Yang et al. 2003). In addition, the effects of partial obstruction of the organ on its mechanical properties were studied in rodents (Liao et al. 2010; Sun et al. 2017), and how these properties changed as a function of obstruction time were also investigated (Zhao et al. 2010; Sun et al. 2018). The effect of partial obstruction on the active behaviour of the small intestine was studied in guinea pigs (Zhao et al. 2011a, b), while Zhao and Gregersen (2015) studied the effect of ageing on the passive material response of the organ in rats.

Several studies investigated the effects of diet on the small intestine: how starvation (Dou et al. 2002b) and re-feeding affects the mechanical properties of the small intestine was evaluated in rats by Dou et al. (2001), how varying amounts of dietary protein affects minks by Chen et al. (2009), the effects of a low-residue (Liu et al. 2017b) and low-fibre (Liu et al. 2017a) diet in rabbits, and the influence of a low-fibre diet on the active mechanical properties in rabbits (Liu et al. 2019). The active properties of the small intestine were considered *ex vivo* in rabbits (Elbrønd et al. 1991; Liu et al. 2019), rats (Ouyang et al. 1996; Zhao et al. 2008, 2013b, 2019), guinea pigs (Zhao et al. 2011a, b), mice (Lu et al. 2012) and pigs (Terry et al. 2011), while no active studies were conducted using human tissue *ex vivo*.

**Fig. 16:**
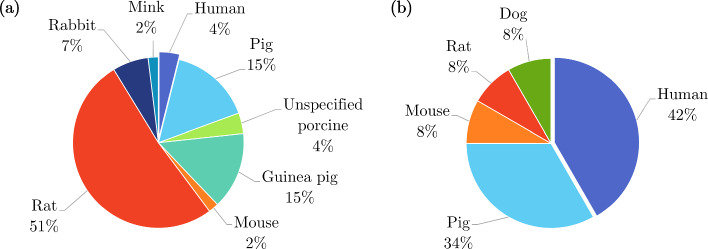
Pie charts indicating the species used in the *ex vivo* experimentation (n=103) **a** and *in vivo* experimentation (n=12) **b** on the small intestine, highlighting, in particular, the proportion of experiments conducted on human tissue

There were a number of studies that looked at the properties of the small intestine *in vivo*, a summary of which can be found in Table 8. Of these, Pedersen et al. (2003), Gregersen et al. (2007) and Gao et al. (2009) evaluated the effect (disease compared to healthy controls) of systemic sclerosis on both the passive and active mechanical properties of the small intestine in humans, and Frøkjær et al. (2007) investigated the active response of the small intestine in patients with type-1 diabetes and compared the observed behaviour to that of healthy controls. Moreover, the active properties of healthy humans and mice were studied *in vivo* by Gao et al. (2003) and Lu et al. (2012), respectively. Figure 16 shows the proportion of each type of tissue used for both the *ex vivo* experimentation (Fig. 16a) and the *in vivo* experimentation (Fig. 16b). The majority of *ex vivo* experiments were conducted using rats, with only 4% on human tissue, while the main proportion of *in vivo* experiments were carried out on humans (42%) closely followed by pigs (34%).

**Table 7:** Summary of *ex vivo* studies on the small intestine

	Species family	Tissue characterisation	Type of test	References
Isotropic	Human	Intact wall	Uniaxial tension	Hosseini and Dunn (2020); Johnson et al. (2020)
			Uniaxial tension (dynamic)	Bourgouin et al. (2012)
	Porcine	Intact wall	Uniaxial tension	Terry et al. (2014); Hosseini and Dunn (2020); Johnson et al. (2020)
			Simple shear (dynamic)	Zhou et al. (2014); Zhang et al. (2014)
			Indentation	Rosen et al. (2008)
			Distension (pressure-CSA)	Jørgensen et al. (1991)
			Tribological test	Zhou et al. (2014)
			Extrusion test	Lyons et al. (2012)
			Zero-stress state	Gao et al. (2000)
		Layer-dependent	Uniaxial tension	Hosseini and Dunn (2020)
			Tribological test	Terry et al. (2011)
	Lagomorph	Intact wall	Distension (pressure-diameter)	Liu et al. (2017b, 2017a, 2019)
			Zero-stress state	Liu et al. (2017b, 2017a, 2019)
	Weasel	Intact wall	Zero-stress state	Chen et al. (2009)
	Rodent	Intact wall	Distension (pressure-diameter)	Chen et al. (2008); Liu et al. (2012); Yang et al. (2012); Zhao et al. (2013b, a, 2017, 2019)
			Distension (pressure-length)	Dou et al. (2006)
			Distension (pressure-CSA)	Storkholm et al. (1995); Vinter-Jensen et al. (1996); Jørgensen et al. (2001)
			Distension (pressure-volume)	Gregersen et al. (1998); Carniel et al. (2014)
			Inflation–extension	Zhao et al. (2008, 2010, 2011a, 2011b)
			Tension of ring specimens	Peck and Gibbs (1987)
			Axial tension of tubular specimens	Zhao et al. (2003b); Radhakrishnan et al. (2005, 2006)
			Zero-stress state	Gregersen et al. (1997); Dou et al. (2001); Zhao et al. (2002); Dou et al. (2002a); Zhao et al. (2002); Dou et al. (2002b); Zhao et al. (2002); Dou et al. (2003); Liao et al. (2003); Yang et al. (2003); Zhao et al. (2003a); Lu et al. (2005); Radhakrishnan et al. (2005); Smith et al. (2005); Radhakrishnan et al. (2006); Dou et al. (2006)
				Sha et al. (2006); Chen et al. (2008); Li et al. (2008); Zhao et al. (2008, 2010); Liao et al. (2010); Zhao et al. (2011a, b); Liu et al. (2012); Zhao et al. (2013b, a); Zhao and Gregersen (2015); Sun et al. (2017); Sokolis (2017); Zhao et al. (2019)
		Layer-dependent	Uniaxial tension	Ouyang et al. (1996)
			Distension (pressure-diameter)	Chen et al. (2008)
			Zero-stress state	Chen et al. (2008)
Anistoropic	Human	Intact wall	Uniaxial tension	Egorov et al. (2002)
	Porcine	Intact wall	Uniaxial tension	Nagaraja et al. (2021); Carvalho et al. (2022)
			Biaxial tension	Terry et al. (2011); Bellini et al. (2011); Terry et al. (2014)
			Pure shear	Davis et al. (2018)
			Distension (pressure-diameter-length)	Gao et al. (2000)
		Layer-dependent	Uniaxial tension	Elbrønd et al. (1991)
	Weasel	Intact wall	Distension (pressure-diameter-length)	Chen et al. (2009)
	Rodent	Intact wall	Distension (pressure-diameter-length)	Dou et al. (2001); Zhao et al. (2002); Dou et al. (2002a, b, 2003); Zhao et al. (2003a); Lu et al. (2005); Liu et al. (2012); Zhao and Gregersen (2015)
			Inflation–extension	Liao et al. (2003); Yang et al. (2003); Liao et al. (2010); Sokolis (2017)
			Inflation–extension–torsion	Sun et al. (2017)
			Zero-stress state	Sun et al. (2018)

**Table 8:** Summary of *in vivo* studies on the small intestine

	Species family	Tissue condition	Type of test	References
Isotropic	Human	Intact wall	Distension (pressure-CSA)	Gao et al. (2003); Pedersen et al. (2003); Gregersen et al. (2007); Gao et al. (2009)
			Distension (pressure-volume)	Gao et al. (2003)
	Porcine	Intact wall	Indentation	Rosen et al. (2008)
			Distension (pressure-CSA)	Jørgensen et al. (1995)
			Contact force test	Terry et al. (2011)
		Layer-dependent	Tribological test	Terry et al. (2011)
	Canine	Intact wall	Distension (pressure-volume)	Shafik (1998)
Anisotropic	Human	Layer-dependent	Distension (pressure-CSA)	Frøkjær et al. (2007)

### Large intestine

Approximately 20% of all *ex vivo* articles collected in the review conducted experimentation on the large intestine (Fig. 13); a summary of these experiments can be found in Table 9. Notably, the effects of a number of diseases on the mechanical behaviour of the large intestine were investigated, including chronic obstruction of the colon in mice which mimics human Hirschsprung’s disease (Hillemeier and Biancani 1990), colitis in rodents (Stidham et al. 2011; Gong et al. 2017; Nair et al. 2018, 2019) and human growth hormone as a potential treatment for this in rats (Christensen et al. 1993), ulcerative colitis in mice (Yang et al. 2009), diabetes in rats (Zhao et al. 2009), Crohn’s disease in humans (Stidham et al. 2011), IBS in rats (Zhao et al. 2019), and cancer in humans (Deptuła et al. 2020). Conditions such as hypertension were also studied in rats (Stewart et al. 2016), and the active response of large intestinal muscle to inflammatory mediators was investigated in both humans and rabbits (Percy et al. 1990). Additionally, the effect of coeliac ganglionectomy on the mechanical properties of the large intestine was evaluated in rats (Ouyang et al. 1996). Yang et al. (2003) looked at the result of EGF treatment over varying periods of time on the mechanical properties of the rat large intestine. Watters et al. (1985) and Massalou et al. (2019a) considered the effects of age and sex on the material behaviour of the large intestine in rats and humans, respectively, and in another study, Watters et al. (1985) looked at the influence of ethnic origin in humans. In terms of the effect of food-intake on the mechanical properties of the intestines, Liu et al. (2017a) investigated the consequence of a long-term low-fibre diet in rabbits.

**Fig. 17:**
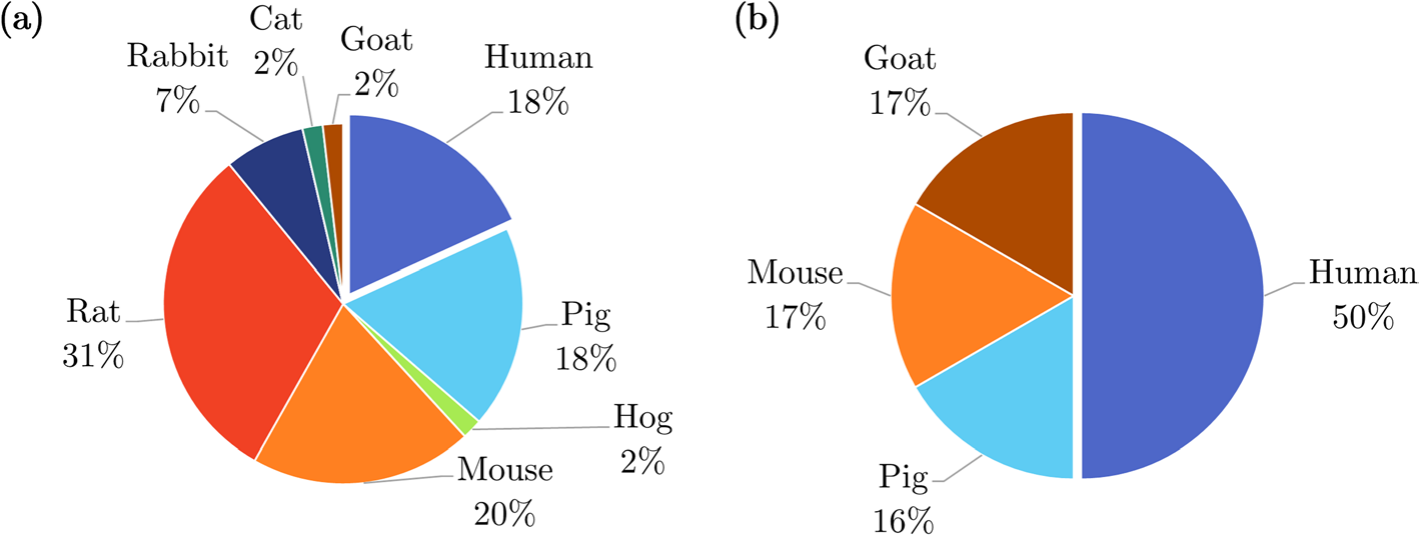
Pie charts indicating the species used in the *ex vivo* experimentation (n = 55) **a** and *in vivo* experimentation (n = 6) **b** on the large intestine, highlighting, in particular, the proportion of experiments conducted on human tissue

As can be seen in Fig. 17a, experiments on rodents, specifically mice and rats, accounted for 51% of the *ex vivo* experimentation on the large intestine, with only 18% conducted using human tissue. Contrarily, half of all *in vivo* experimentation regarding the large intestine was carried out on humans, as shown in Fig. 17b. A summary of the *in vivo* experiments conducted on the colon can be found in Table 10. Of these experiments, Petersen et al. (2001) assessed the relationship between pain during distension of the large intestine and its stress–strain response in healthy human subjects, while Drewes et al. (2001) studied the difference in pain during large intestinal distension, and its associated biomechanical parameters, between patients with IBS and healthy human controls.

In terms of the active properties of the large intestine, *ex vivo* experimentation was carried out on rabbit (Pescatori et al. 1979; Percy et al. 1990), human (Gill et al. 1986; Percy et al. 1990), cat (Merlo and Cohen 1988) and rat (Ouyang et al. 1996; Zhao et al. 2019) tissue, and *in vivo* experimentation was conducted on humans (Bharucha et al. 2001). For further understanding of the active behaviour of the large intestine from a mechanical perspective, readers are referred to the literature review of Bhattarai et al. (2022a).

**Table 9:** Summary of *ex vivo* studies on the large intestine

	Species family	Tissue condition	Type of test	References
Isotropic	Human	Intact wall	Uniaxial tension (dynamic)	Massalou et al. (2019a)
			Shear rheometry	Deptuła et al. (2020)
			Tension of ring specimens	Watters et al. (1985)
			Elastography	Stidham et al. (2011)
		Layer-dependent	Uniaxial tension	Gill et al. (1986)
	Porcine	Intact wall	Zero-stress state	Patel et al. (2018)
	Caprine	Intact wall	Uniaxial compression	Higa et al. (2006)
	Feline	Layer-dependent	Uniaxial tension	Merlo and Cohen (1988)
	Lagomorph	Intact wall	Distension (pressure-diameter)	Liu et al. (2017a)
			Zero-stress state	Liu et al. (2017a)
	Rodent	Intact wall	Uniaxial tension	Gong et al. (2017)
			Indentation	Stewart et al. (2016)
			Distension (pressure-diameter)	Itasaka et al. (1992); Lu et al. (2012); Zhao et al. (2019)
			Tension of ring specimens	Watters et al. (1985); Hillemeier and Biancani (1990)
			Elastography	Stidham et al. (2011); Nair et al. (2018, 2019)
			Zero-stress state	Gao and Gregersen (2000); Yang et al. (2003, 2009); Zhao et al. (2009); Sokolis et al. (2011); Siri et al. (2019a); Zhao et al. (2019)
		Layer-dependent	Uniaxial tension	Ouyang et al. (1996)
			Distension (pressure-diameter)	Itasaka et al. (1992)
Anisotropic	Human	Intact wall	Uniaxial tension	Egorov et al. (2002); Christensen et al. (2015)
			Uniaxial tension (dynamic)	Massalou et al. (2016, 2019b)
		Layer-dependent	Uniaxial tension	Percy et al. (1990)
	Porcine	Intact wall	Uniaxial tension	Ciarletta et al. (2009); Carniel et al. (2014b, a); Christensen et al. (2015)
			Biaxial tension	Puértolas et al. (2020); Bhattarai et al. (2022a, b)
			Pure shear	Davis et al. (2018)
			Simple shear	Ciarletta et al. (2009)
			Inflation–extension	Patel et al. (2018)
		Layer-dependent	Uniaxial tension	Carniel et al. (2014b)
	Lagomorph	Intact wall	Uniaxial tension	Pescatori et al. (1979)
		Layer-dependent	Uniaxial tension	Percy et al. (1990)
	Rodent	Intact wall	Biaxial tension	Siri et al. (2019a)
			Distension (pressure-diameter-length)	Gao and Gregersen (2000); Yang et al. (2009); Zhao et al. (2009)
			Inflation–extension	Sokolis et al. (2011); Sokolis and Sassani (2013)
		Layer-dependent	Biaxial tension	Siri et al. (2019b)
			Zero-stress state	Siri et al. (2019b)

**Table 10:** Summary of *in vivo* studies on the large intestine. It should be noted that all the studies referenced here studied the behaviour of the large intestine in just one direction (isotropic)

Species family	Tissue characterisation	Type of test	References
Human	Intact wall	Distension (pressure-CSA)	Drewes et al. (2001); Petersen et al. (2001)
		Distension (pressure-volume)	Bharucha et al. (2001)
Porcine	Intact wall	Indentation	Rosen et al. (2008)
Caprine	Intact wall	Uniaxial compression	Higa et al. (2007)
Rodent	Intact wall	Distension (pressure-diameter)	Lu et al. (2012)

### Rectum

**Fig. 18:**
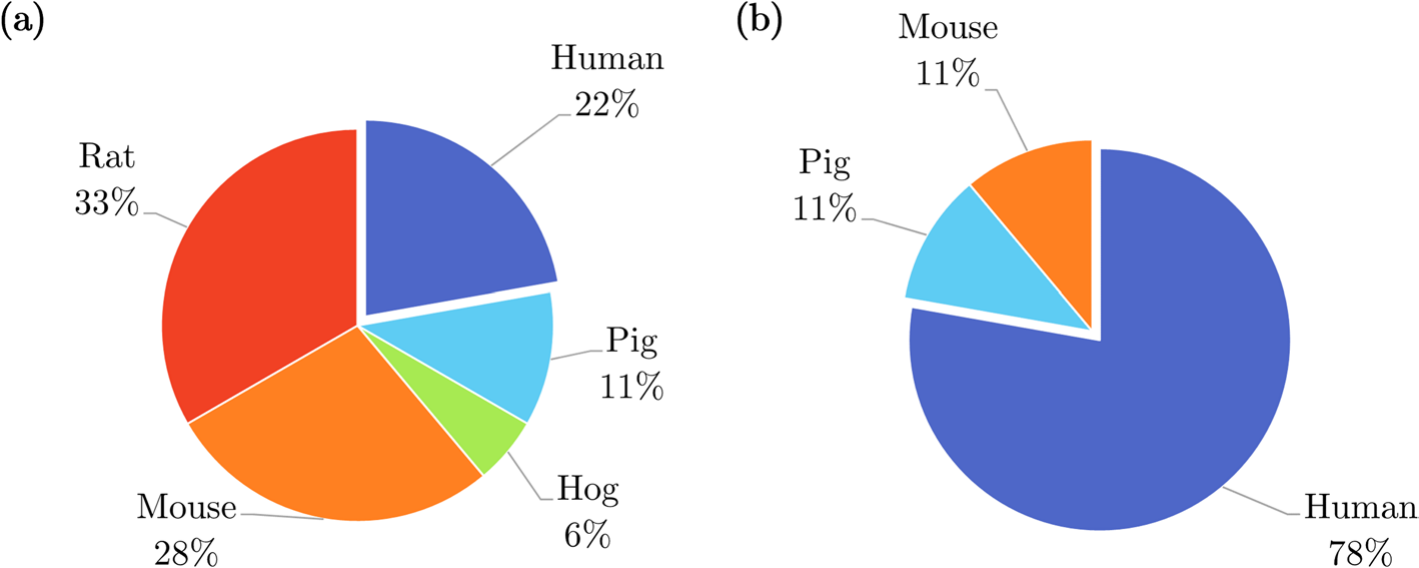
Pie charts indicating the species used in the *ex vivo* experimentation (n = 18) **a** and *in vivo* experimentation (n = 9) **b** on the rectum, highlighting, in particular, the proportion of experiments conducted on human tissue

The rectum had the least amount of *ex vivo* mechanical experimentation compared to the other GI organs (Fig. 13), a summary of which can be found in Table 11. Notable studies included those by Watters et al. (1985) who looked at the influence of sex and age on the material behaviour of the rectum in rats; Glavind et al. (1993) who conducted experimentation in regard to the active properties of the human rectum’s muscle layer; Gregersen et al. (2002) who studied how the rectum of mice was affected by irradiation; Yang et al. (2003) who evaluated the change in mechanical properties experienced when growth of the rat rectum was induced by EGF; and Brunenieks et al. (2017) who investigated the effect of obstructed defecation syndrome on the biomechanical properties of the human rectal wall, comparing the abnormal tissue extracted from surgical resection to tissue excised from healthy humans post-mortem. Figure 18a shows that most *ex vivo* experimentation on the rectum was carried out using rodent tissue (specifically, mice and rats), comprising 61% of the total number of experiments conducted.

It can be seen in Fig. 18b that the vast majority of *in vivo* experimentation of the rectum was conducted on humans. Of these *in vivo* experiments, of which a summary can be found in Table 12, a few investigated the effects of different conditions. For instance, Arhan et al. (1978) studied the difference in viscoelastic behaviour of the rectal wall between patients with Hirschsprung’s disease and healthy, age-matched controls; Lundby et al. (1999) looked at the effect of age on the mechanical properties of the rectum in mice; and Petersen et al. (2001) conducted experimentation to assess the biomechanical behaviour of the human rectum, studying how the pain felt by the volunteer during distension was associated with the tissue’s stress–strain response. The same group then went on to look at how the mechanical response and pain differed during distension before and after smooth muscle relaxation (Petersen et al. 2003). Furthermore, Drewes et al. (2001) investigated the difference in rectal mechanical parameters and levels of pain between patients with IBS and healthy human controls, and in another study evaluated again the relation between pain and biomechanical properties of the rectum but this time in patients with ulcerative colitis (Drewes et al. 2006), comparing their results against healthy controls.

**Table 11:** Summary of *ex vivo* studies on the rectum

	Species family	Tissue condition	Type of test	References
Isotropic	Human	Intact wall	Uniaxial tension	Brunenieks et al. (2017)
		Layer-dependent	Uniaxial tension	Glavind et al. (1993)
	Porcine	Intact wall	Uniaxial tension	Pedro et al. (2014)
			Simple shear	Qiao et al. (2005)
			Uniaxial compression	Qiao et al. (2005)
	Rodent	Intact wall	Tension test of ring specimens	Watters et al. (1985)
			Zero-stress state	Gao and Gregersen (2000); Gregersen et al. (2002); Yang et al. (2003); Sokolis et al. (2011); Siri et al. (2019a)
Anisotropic	Human	Intact wall	Uniaxial tension	Rubod et al. (2012); Christensen et al. (2015)
	Porcine	Intact wall	Uniaxial tension	Qiao et al. (2005); Christensen et al. (2015)
	Rodent	Intact wall	Biaxial tension	Siri et al. (2019a)
			Distension (pressure-diameter-length)	Gao and Gregersen (2000)
			Inflation–extension	Sokolis et al. (2011)
		Layer-dependent	Biaxial tension	Siri et al. (2019b)
			Zero-stress state	Siri et al. (2019b)

**Table 12:** Summary of *in vivo* studies on the rectum

	Species family	Tissue condition	Type of test	References
Isotropic	Human	Intact wall	Distension (pressure-diameter)	Arhan et al. (1978)
			Distension (pressure-CSA)	Dall et al. (1993); Drewes et al. (2001); Petersen et al. (2001, 2003); Drewes et al. (2006)
	Porcine	Intact wall	Distension (pressure-CSA)	Dall et al. (1991)
	Rodent	Intact wall	Distension (pressure-CSA)	Lundby et al. (1999)
Anisotropic	Human	Intact wall	Distension (pressure-CSA-arc length)	Frøkjær et al. (2005)

### Experimental particulars

In this section of the review findings, we will focus on the particulars of the experiments including which experiments involved investigation of the tissue’s time-dependent behaviour (Sect. 4.6.1), whether preconditioning of the sample was performed prior to data collection (Sect. 4.6.2), if, for the *ex vivo* experimentation, the tests were carried out in a saline solution bath (Sect. 4.6.3), and whether the studies conducted histological analysis alongside their mechanical experimentation to provide information on the microstructural components of the tissue and how they might influence its stress–strain behaviour (Sect. 4.6.4).

#### Time-dependent behaviour

**Fig. 19:**
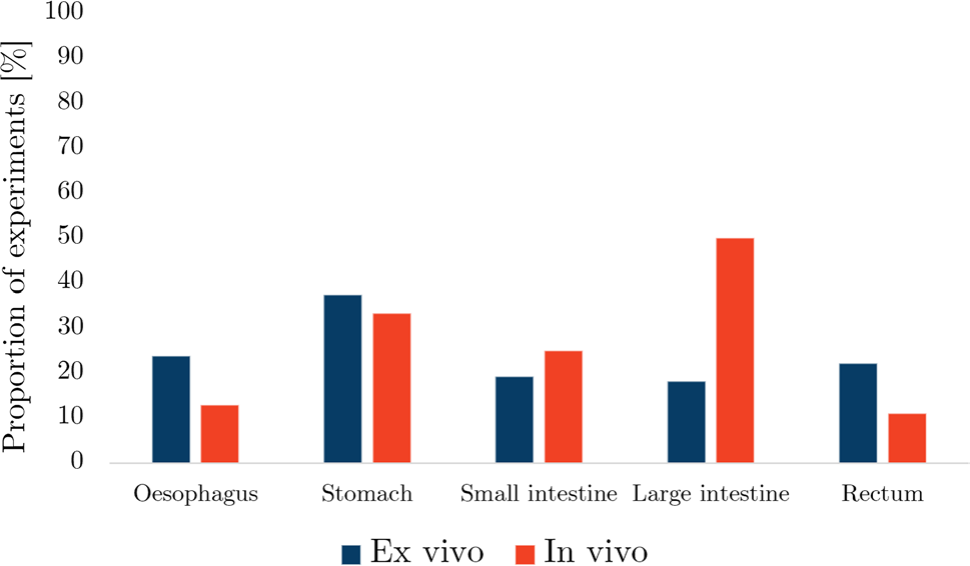
Proportion of studies for each organ, specified according to *ex vivo* and *in vivo* experimentation, that investigated the time-dependent behaviour of the tissue

Soft tissues often present as viscoelastic materials (Righi and Balbi 2021); this means that relaxation and creep can be seen in their material response, and, thus, that their mechanical behaviour is time-dependent. Some of the studies included in this review investigated the time-dependent behaviour of the GI organs, a summary of which can be found in Table 13. The proportion of experiments for each organ in which their material response was considered as a function of time is illustrated in Fig. 19.

**Table 13:** Summary of studies that considered the time-dependent behaviour of the GI tissues

Tissue	Species family	References	
		*Ex vivo*	*In vivo*
Oesophagus	Human	Durcan et al. (2022a, 2022b)	
	Porcine	Tay et al. (2006); Yang et al. (2006c, b); Aho et al. (2016a, b); Lin et al. (2017)	Tay et al. (2006)
	Caprine	Taira et al. (2014)	
	Lagomorph		Gregersen et al. (1988)
	Rodent	Wareham and Whitmore (1982); Gregersen et al. (1999); Yang et al. (2004); Liao et al. (2009); Jiang et al. (2014, 2017, 2019)	Goyal et al. (1971)
Stomach	Human	Lim et al. (2009); Carniel et al. (2020); Toniolo et al. (2022)	
	Porcine	Jia et al. (2015); Salmaso et al. (2020); Borsdorf et al. (2021); Tomalka et al. (2017); Julie et al. (2022)	Rosen et al. (2008)
Small intestine	Human	Egorov et al. (2002); Johnson et al. (2020)	Gao et al. (2003); Gregersen et al. (2007)
	Porcine	Jørgensen et al. (1991); Rosen et al. (2008); Lyons et al. (2012); Zhou et al. (2014); Zhang et al. (2014); Johnson et al. (2020)	Rosen et al. (2008)
	Lagomorph	Liu et al. (2017a)	
	Rodent	Storkholm et al. (1995); Gregersen et al. (1998); Zhao et al. (2003b); Smith et al. (2005); Zhao et al. (2008); Yang et al. (2012); Carniel et al. (2014); Zhao et al. (2017)	Liao et al. (2012)
Large intestine	Human	Watters et al. (1985); Massalou et al. (2019b); Deptuła et al. (2020)	Bharucha et al. (2001)
	Porcine	Ciarletta et al. (2009)	Rosen et al. (2008)
	Caprine	Higa et al. (2006)	Higa et al. (2007)
	Lagomorph	Liu et al. (2017a)	
	Rodent	Watters et al. (1985); Stewart et al. (2016); Siri et al. (2019a, b)	
Rectum	Human		Arhan et al. (1978)
	Rodent	Watters et al. (1985); Siri et al. (2019a, b)

#### Preconditioning

**Fig. 20:**
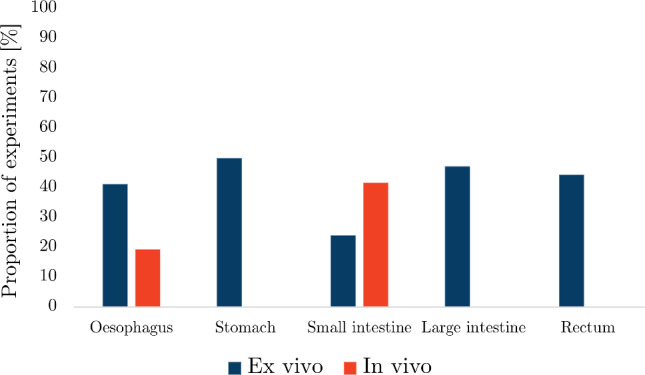
Proportion of studies for each organ, specified according to *ex vivo* and *in vivo* experimentation, that preconditioned the tissue

Preconditioning is the process of “conditioning” a sample before collecting data in regard to its material response and involves loading and unloading the sample successively for a predetermined number of cycles. The process came about through the study of polymers, which behave in a similar way to soft tissues in that they are highly elastic, usually possess viscous qualities and can exhibit history-dependent behaviour. Preconditioning of polymers is to remove the Mullins effect: a purely history-dependent softening of the material that depends on the previous maximum strain that it has been subjected to (Dorfmann and Ogden 2004). With soft tissues, the equivalent term is stress-softening. It was once thought that preconditioning reduced the influence of soft tissues’ time-dependent, i.e. viscous, properties, however, through research of the myocardium by Emery et al. (1997), it was established that it has mainly an effect on their history-dependent response. This was confirmed as well for the guinea pig small intestine by Gregersen et al. (1998). Therefore, the preconditioning process for soft tissues results in reducing history-dependent effects on their behaviour, as well as some time-dependent effects, which tend to plateau after a minimum of three repeated cycles. Figure 20 shows the proportion of studies evaluated in this review that preconditioned the tissue before collecting their results, for both *in vivo* and *ex vivo* experiments.

#### Saline solution bath

**Fig. 21:**
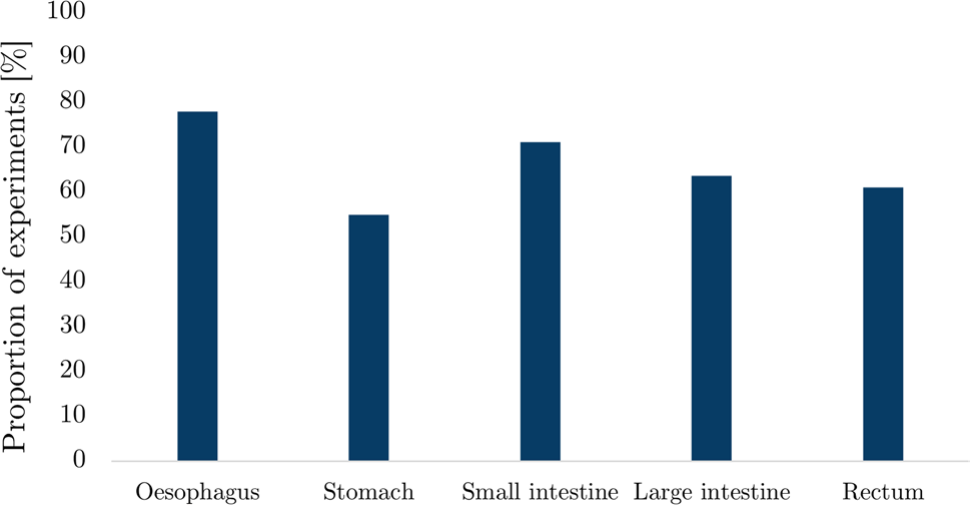
Proportion of *ex vivo* studies for each organ that conducted the experiments within a salt solution bath

As previously mentioned in Sect. 3.1, for *ex vivo* mechanical experimentation, measures are often taken to simulate a physiological environment. The main method for achieving this is by conducting experiments on samples immersed in a chamber (or bath) filled with a salt solution. This is done to prevent dehydration of the soft tissue, which has been found to cause alteration to their mechanical properties (Nicolle and Palierne 2010). Sometimes these chambers are thermoregulated so that the temperature of the tissue can be maintained at internal body temperature (37°C) throughout testing. As can be seen in Fig. 21, the majority of *ex vivo* experiments considered in this review were performed using a saline solution bath, the organ with the highest proportion being the oesophagus with 78%. Almost all *ex vivo* studies stored their tissue specimens in some variety of salt solution between tests; however, Fig. 21 only shows the percentage of those which performed their tests in a solution bath. The other studies, e.g. the remaining 28% of the oesophageal experiments, often kept the samples moist by alternative means such as spraying the samples during testing; however, Nicolle and Palierne (2010) concluded that the best method to prevent dehydration of soft tissue samples is by conducting the tests in a saline bath. The types of salt solutions that were used in the experimental studies on the GI tissues included physiological saline, phosphate-buffered saline (PBS) and Krebs solution, which were sometimes aerated with oxygen and carbon dioxide.

#### Histology

**Fig. 22:**
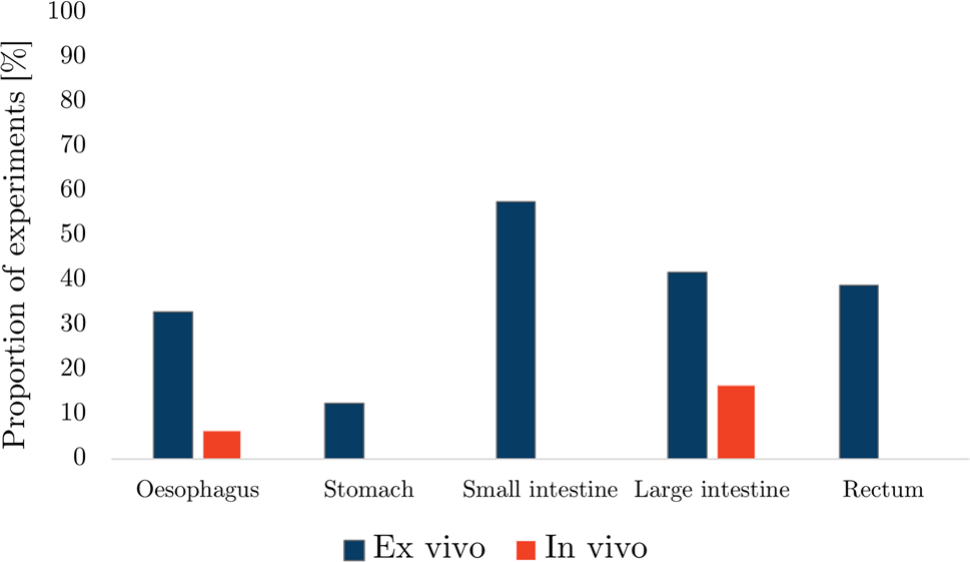
Proportion of studies for each organ, specified according to *ex vivo* and *in vivo* experimentation, that investigated the histological composition of the tissue alongside their mechanical tests

As previously briefly discussed in Sect. 1, the microstructural components of soft tissues influence their macrostructural behaviour. Histological analysis provides a well-established means to investigate the various microstructural features of tissues, the images from which can be used to establish the prevalence (fraction) and orientation of their collagen, elastin and muscle fibres (Fan et al. 2005). The analysis is carried out by removing a very thin slice of a tissue sample, putting the slices on a slide and then using different stains to highlight different microstructural features (Durcan et al. 2022a; Girard et al. 2019). Finally, images are taken which can then be post-processed and analysed to establish the fraction and orientation of the aforementioned fibres. This information can help to discuss reasons for the experimentally observed behaviour and potentially deduce their more specific affect (for example, by artificially increasing or decreasing the fraction of fibres and using the histological images to quantify the change), and inform micromechanical constitutive modelling (Masri et al. 2018). Figure 22 shows the proportion of experiments that conducted histological inspection alongside their biomechanical investigation. Histological analysis was considered here because it is the most prevalent and traditional means of assessing the microstructure of soft tissues; however, for an outline of more modern techniques such as second-harmonic generation (SHG) microscopy and optical-based analysis, readers are referred to Siri et al. (2020) and Goth et al. (2016), respectively.

## Discussion

The review findings showed that the GI tissues of a number of different species were tested using an array of experimental approaches. Here, some of the experimental aspects will be discussed in more detail.

### *In vivo* vs. *ex vivo*

The main drive of mechanically testing human soft tissues is to establish their material behaviour in the context of their natural environment, i.e. for the GI tract as digestive organs inside the body, for which *in vivo* studies provide more realistic behaviour being that the tissue is still alive and perfused with blood (Lim et al. 2009). Other aspects such as the internal temperature, moisture levels and structural integrity of the organ are also maintained during *in vivo* testing (Dargar et al. 2016). The use of a thermoregulated saline bath can be used for *ex vivo* experimentation in an attempt to control the temperature and moisture variables; however, the tissue is still deceased and will not have exactly the same mechanical properties as it would *in vivo* due to phenomena such as rigour mortis and the relaxation of residual stresses (Dargar et al. 2016). The structural integrity can be maintained during *ex vivo* experiments such as distension and inflation–extension tests; however, the organ being tested has still been detached from its natural position and the connective tissue holding the organ in place has been cut; therefore, aspects such as its interaction with surrounding organs or structures are not included in its characterisation (Marie et al. 2019). Despite this, the *in vivo* experimentation carried out on the GI organs were mostly distension tests where the behaviour was characterised in only one direction and a homogeneous tissue wall was assumed, while testing *ex vivo* allows for a wider variety of experimental tests and the more complex behaviour of the organ to be investigated. Furthermore, the force–displacement measurements obtained during *ex vivo* experimentation can be much more accurate compared to those from *in vivo* experiments, for which measurements are often obtained from relatively low-resolution imaging techniques, while also potentially being disrupted by movement and breathing of the subject (Dargar et al. 2016), thus increasing the error associated with the mechanical properties determined.

In addition, the deformation of the tissue in supraphysiological loading domains, such as is the case in surgery (Lim et al. 2009) or road traffic accidents (Massalou et al. 2016), cannot be carried out *in vivo* as this may cause irreversible damage to a subject that is still living, whereas *ex vivo* experimentations allow for the rupture points and dynamic properties of the tissues to be established because the organ is no longer required (Durcan et al. 2022b; Massalou et al. 2019b). The ethical constraints associated with *in vivo* testing for both animals and humans are much greater than for *ex vivo* experimentation due to the pain, discomfort and damage the tests might cause to a living subject. Furthermore, data that are collected from a living subject often have more noise associated with it compared to *ex vivo* testing due to the movement caused by the beating heart and respiration (Rosen et al. 2008). In terms of layer-dependent properties of a GI tissue, these are usually more easily established *ex vivo* by separating the layers, normally the two main layers (mucosa-submucosa and muscular layer), and testing them individually. However, recently, Dargar et al. (2019) used compression elastography to determine the layer-dependent properties of porcine stomach tissue *in situ*, while the animal was still alive and was able to characterise the submucosa, mucosa and muscular layers individually up to a strain of 20%, i.e. beyond the linear elastic regime. This provides hope for the development of an experimental technique that allows for a similar characterisation completely *in vivo*. Moreover, residual strains within the GI organs are traditionally established *ex vivo*. However, methods to quantify them *in vivo* are being developed for arterial tissue (Donmazov et al. 2015; Aggarwal et al. 2016), which can be applied to the GI organs due to their similar anatomical structure.

There are benefits and limitations to both *in vivo* and *ex vivo* experimentation; however, *in vivo* testing provides a more realistic understanding of the behaviour of soft tissues in the conditions we are interested in. Therefore, effort should be made to further develop *in vivo* mechanical characterisation techniques, such as ultrasound elastography (Dargar et al. 2019), that allow for the layer-dependent properties to be established in a direction-dependent manner, as well as the organ’s internal residual stresses and strains. *Ex vivo* experimental characterisation should still be carried out for the higher end of large strain deformations, i.e. supraphysiological loading, of human tissues.

### Organs tested

Out of all the experimental articles, the oesophagus was the organ investigated the most, with a total of 82 articles collected in this review (Table 2). The tissue studied the least was the rectum for which 21 articles were found in regard to its biomechanical characterisation, closely followed by the stomach with 28 articles. While the small intestine had a greater number of *ex vivo* articles than the oesophagus, the oesophagus had, overwhelmingly, the highest number of *in vivo* articles which contributed towards the organ having the most experimental articles overall. The high number of *in vivo* tests compared with the other organs could be due to the anatomical position of the oesophagus in that it is easily accessible for biomechanical measurements using a probe inserted through the mouth. The same can be said for the rectum, where the number of *in vivo* articles is almost the same as the *ex vivo* articles, a relationship not seen for any of the other organs, for which the number of *ex vivo* articles is much higher than the *in vivo* studies, particularly for the stomach, small intestine and large intestine. This is also thought be due to the more accessible position of the rectum where in which a probe can be simply positioned through the anus.

### Species tested

The findings show that animal tissue was used far more prevalently than human tissue for mechanical testing of the GI tissues: out of the articles considered, human tissue was investigated in 20% of the studies on the oesophagus, 21% of the studies on the stomach, 8% of the studies on the small intestine, 21% of the studies on the large intestine and in 41% of the studies on the rectum. This could be due to the fact that animals/animal tissues are a lot more accessible and are associated with fewer ethical constraints compared to testing with humans/human tissues. As mentioned in Sect. 5.2, the greater proportion of human studies on the rectum is thought to be due to it being a more easily accessible GI organ (along with the oesophagus) when conducting studies on live humans (*in vivo*).

For applications within medicine where the material properties of the tissue will be used quantitatively, such as to provide force feedback to a surgeon using a haptic simulator (Chakravarthy et al. 2014), biomechanical data from human tissue should be used. However, there are benefits to using animal tissue, particularly for the investigation of diseased states, and discussing this data qualitatively in regard to the human organ. The greatest benefit may be demonstrated through the use of mice or rat models. These animals are able to be grown in a very controlled environment, where their age, diet, living conditions, etc., can be decided and closely monitored. This allows for the environmental factors that influence the mechanical behaviour of the tissue, and which contribute to variability in the data, to be controlled and recorded, producing more reproducible data than say between different human specimens. Additionally, there are many rat and mice models that exist to simulate different human diseases, such as type-1 and type-2 diabetes, IBS, and Hirschsprung’s disease. Therefore, testing of these animals allows for a highly controlled investigation of the effects of disease on the mechanical properties of the organs. However, quantitatively, the mechanical results of experiments conducted on animals tissues will not be the same as for human tissues as aspects such as size, tissue structure and digestive demands differ, and so these results should not be used to determine the material parameters for models that will be used in medicine unless no human experimental data are available. Porcine tissue is often chosen due to porcine having a digestive system close to that of humans (Salmaso et al. 2020), however, when comparing between human and porcine data, there are still significant differences between their mechanical properties and so, ideally, data from porcine tissue should not be used directly for applications within medicine (Christensen et al. 2015).

### Sample size

In addition to providing a better control of experimental design than with human specimens, animal specimens often offer the possibility to test a larger sample size, making the final results more robust. Either it is difficult to obtain human volunteers for *in vivo* tests, especially for the GI tract which can bring, compared to testing organs such as the skin, more discomfort, or there is a limited availability of human cadavers for *ex vivo* testing. For both *in vivo* and *ex vivo* testing with humans, there are ethical constraints that must be considered. For *in vivo* mechanical testing, informed consent must be given and the study protocol must ensure that no unnecessary harm is caused to the patient or volunteer. For *ex vivo* experimentation, the tissue obtained from the human cadavers must not be wasted and should only be completed when a clear experimental methodology is established: knowing the purpose of each test and its aims. With animals, these ethical constraints are still present but are more relaxed than with humans. High-quality *in silico* models could reduce the need for animal and human experimentation, which is always preferable from an ethical perspective.

### Anisotropy

Approximately half of the *ex vivo* experiments and almost all of the *in vivo* experiments referenced in this review studied the mechanical properties of their respective tissues in only one direction, usually the circumferential direction. However, from the work of Brasseur et al. (2007), it can be seen how the behaviour in the longitudinal direction affects the efficiency of peristalsis within the GI tract and thus the function of the organs. In addition, many studies have found a discrepancy between the longitudinal and circumferential directions in terms of material response, commonly attributed to the arrangement of fibres such as collagen and elastin in the tissue walls (Siri et al. 2020). Therefore, direction-dependent behaviour should be considered in future experimental investigations, particularly for *in vivo* studies for which anisotropic studies are lacking (Tables 4, 6, 8, 10, 12).

### Layer-dependency

**Fig. 23:**
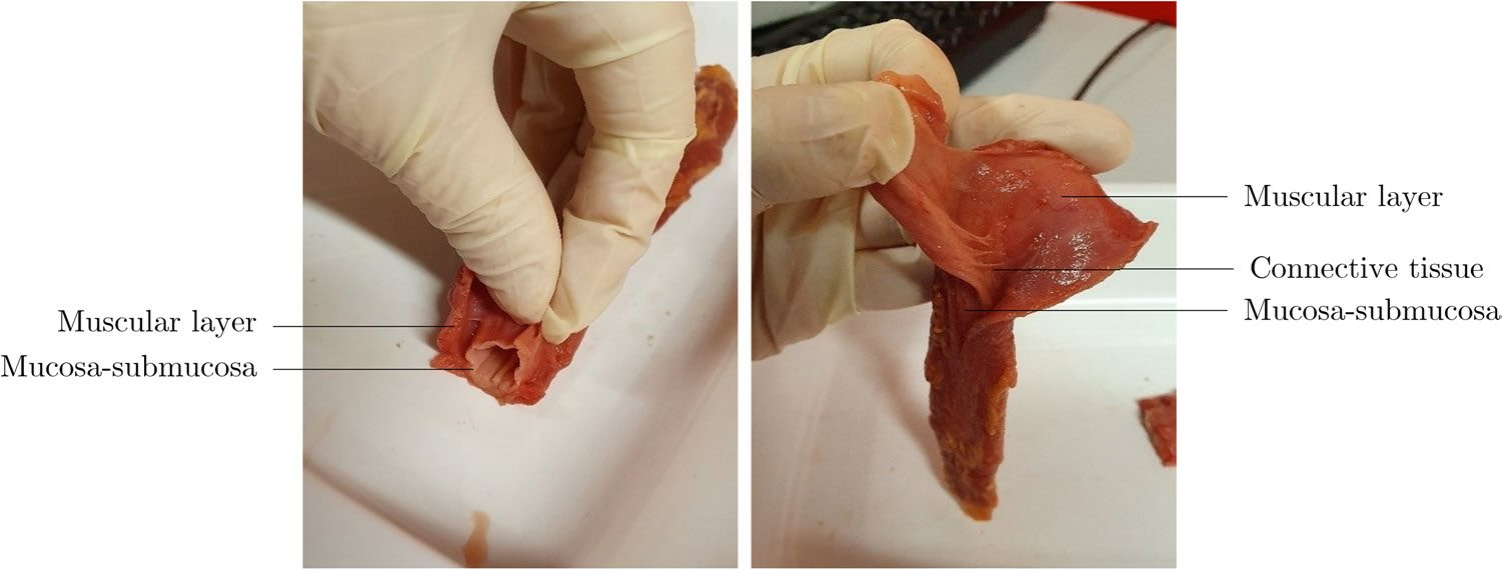
The two main layers of the human oesophagus attached by relatively loose connective tissue. Figure modified from Durcan et al. (2022a)

Those who studied the intact wall of the GI organs assumed the mechanical properties in the radial direction to be homogeneous. However, layer-dependent studies show this not to be the case, with the varying amount of microstructural components, namely collagen and elastin, being the main hypothesis as to why the material behaviour of the layers differ (Siri et al. 2020). It can be seen that the oesophagus has a higher proportion of layer-dependent studies compared to the other tissues. This is due to the oesophagus being the only visceral organ, which can be relatively easily separated into its two main layers (the mucosa-submucosa and the muscularis propria) after explantation (Payan and Ohayon 2017). This can be seen in Fig. 23, which shows that the connective tissue attaching the two main layers of the human oesophagus together is loose, making the layers straightforward to dissect (Durcan et al. 2022a). For the small intestine, it was found in the study by Sokolis (2017) that “preliminary attempts to dissect the layers were not successful”. Some have been successful using micro-dissection; however, since the layers of the GI organs apart from the oesophagus are tightly bound, it may be hard to ensure that no damage has been incurred to any of the layers.

Whether the layer-dependent properties of the organ are considered depends on the application of the experimental work. For instance, for FE modelling of the interaction of a GI organ and a stent, a layer-dependent model will help to understand how the pressure exerted by the stent is supported by each of the layers. However, if the aim is to study the properties of the organ wall under dynamic loading for use in FE models that investigate the impact of blunt trauma, for instance during road traffic collisions, the layer-dependent properties may provide too much detail for the application (Massalou et al. 2016, 2019b, a; Ruan et al. 2003). Nevertheless, many studies show large differences between mechanical behaviour of the different layers, and their influence on the overall function of the organ should be considered to provide a more complete biomechanical understanding of the GI tract (Yang et al. 2007a).

### Preconditioning

Preconditioning is a technique first employed in the characterisation of polymers to reduce the influence of history-dependent, and some time-dependent, effects on the recorded behaviour of a material, making the results more stable and repeatable (Gregersen et al. 1998; Rosen et al. 2008). Within biomechanics, preconditioning is used both *in vivo* and *ex vivo*. Its use, though, is controversial. On the one hand, it makes the behaviour of soft tissues more consistent so the observed material response between different samples and subjects is less variable, however, on the other hand, in many of the applications in which the biomechanics of soft tissues are of interest, their behaviour during the first cycle is the one of most importance, for instance, during normal physiological loading (Bauer et al. 2020), surgery (Rosen et al. 2008), blunt trauma (Johnson et al. 2020) and endoscopy (Carniel et al. 2014b). In these situations, for example, the tissue is not preconditioned before the stomach wall is passively stretched by its contents, or surgical tools manipulate and cut the large intestine. It has even been found that with the rat oesophagus, the material properties of the wall return to what they were before stretching once muscle activation has occurred, i.e. the stress-softening of the wall is reversed during peristalsis (Jiang et al. 2014, 2017, 2019), therefore suggesting that the first-cycle behaviour is the one most often of main interest. In future experimentation, it may be best to quantify both the initial material response and the behaviour after preconditioning as this provides experimental data to be used in the aforementioned applications, as well as information on the history- and time-dependent behaviour of the GI organs (Weizel et al. 2022).

### Limitations of the review

In this review, sphincters of the GI tract have not be included. For comprehensive characterisation of the GI tract, these sphincters would have to be considered and also modelled *in silico* if the application requires. Additionally, an experimental aspect that has not been extensively discussed here is the different methods for strain measurement used in the characterisation of the GI tissues. These can include digital image correlation (DIC) (Khoo et al. 2016), image analysis (Huang et al. 2019), and extensometers within the testing machine (Durcan et al. 2022b). Another aspect that has not been highlighted in the review is the investigation of the plasticity and damage mechanisms of the GI tissues. To increase the complexity of a constitutive model and for specific applications, such as modelling the perforation of a tissue, these irreversible processes should be considered. Moreover, only one database was used, and although particular care was taken to add any articles known by the authors not found in the PubMed search, some experimental studies may have been missed and therefore may not be included in this review.

In the interest of brevity, not all the articles presented in the tables were mentioned in the text, with only those that studied something more than the passive behaviour of normal tissue being highlighted. Furthermore, a comparison of the numerical values of mechanical properties presented in each article has not been carried out. This was due to the large number of articles collected in the review, and the complex nature of comparing numerical values obtained from experiments carried out in different loading modes and with different protocols (such as strain rate and sample dimensions). Instead, the aim of this review was to provide the reader with an overview of the experiments that have been performed on their organ of interest, from which they may either obtain experimental data for a specific loading mode, or perform their own more in-depth analysis and comparison of the current understanding of the organ’s mechanical properties.

## Conclusion

This review was written with the aim to consolidate the mechanical experimentation that has been conducted on the GI tract, to highlight what is missing in the literature in terms of the characterisation of the GI organs, and to be used by readers to inform their own experimental choices or to provide a reference of experimental data for their own analysis and/or constitutive and FE modelling. For the latter application, experimental data can be retrieved for a certain GI organ and type of test, with the test condition (*in vivo* or *ex vivo*), direction-dependency (isotropic or anisotropic) and layer-dependency (intact wall or layer-dependent) also being specified.

In terms of *ex vivo* experimentation, there are little data regarding the human oesophagus and small intestine, with no *ex vivo* active studies being conducted on the small intestine from humans. For *in vivo* mechanical characterisation, no studies included in this review involved experimentation of the human stomach, with only three studies being carried out in total on the stomach *in vivo*. Furthermore, very few *in vivo* characterisations involved determination of the layer-dependent properties of the GI tract. Overall, there is a lack of time-dependent studies on the GI organs, particularly for human tissue with only 4% of all the *ex vivo* articles considering the tissues’ viscoelastic properties and 2% investigating the time-dependent behaviour of human tissue *in vivo*. Moreover, very few studies investigated the shear properties of the tissues, and there were no studies that considered the GI tract’s residual stresses and strains *in vivo*. Compared to the other organs, there was considerably less experimentation conducted on the rectum. Therefore, a focus should be applied to characterising the more complex aspects of the GI organs’ mechanical behaviour using human tissue, ideally *in vivo*, including their layer-dependent, anisotropic, viscoelastic, shear and active properties, as well as their residual stresses and strains. Experimentation should be particularly focused on the stomach and rectum, for which data are lacking overall.

## Abbreviations

GI: Gastrointestinal
H&E: Haematoxylin and eosin
GK: Goto-Kakizaki
FE: Finite element
CFD: Computational fluid dynamics
FSI: Fluid–structure interactions
CSA: Cross-sectional area
EGF: Epidermal growth factor
FCP: Functional chest pain
IBS: Irritable bowel syndrome
PBS: Phosphate-buffered saline
SHG: Second-harmonic generation
DIC: Digital image correlation.
